# Hippocampal metabolic subregions and networks: Behavioral, molecular, and pathological aging profiles

**DOI:** 10.1002/alz.13056

**Published:** 2023-04-04

**Authors:** Somayeh Maleki Balajoo, Simon B. Eickhoff, Shahrzad Kharabian Masouleh, Anna Plachti, Laura Waite, Amin Saberi, Mohamed Ali Bahri, Christine Bastin, Eric Salmon, Felix Hoffstaedter, Nicola Palomero-Gallagher, Sarah Genon

**Affiliations:** 1 Institute of Systems Neuroscience, Heinrich Heine University Duesseldorf, Duesseldorf, Germany; 2 Institute of Neuroscience and Medicine (INM-7), Research Centre Juelich, Juelich, Germany; 3 Danish Research Centre for Magnetic Resonance, Centre for Functional and Diagnostic Imaging and Research, Copenhagen University Hospital Hvidovre, Hvidovre, Denmark; 4 Otto Hahn Research Group for Cognitive Neurogenetics, Max Planck Institute for Human Cognitive and Brain Sciences, Leipzig, Germany; 5 GIGA-Cyclotron Research Centre-In Vivo Imaging, University of Liège, Liège, Belgium; 6 Psychology and Cognitive Neuroscience Research Unit, University of Liège, Liège, Belgium; 7 Department of Neurology, University Hospital of Liège, Liège, Belgium; 8 Institute of Neuroscience and Medicine (INM‑1), Research Centre Juelich, Juelich, Germany; 9 Department of Psychiatry, Psychotherapy and Psychosomatics, Medical Faculty, RWTH Aachen University, Aachen, Germany; 10 Cécile and Oskar Vogt Institute for Brain Research, Heinrich Heine University Duesseldorf, Duesseldorf, Germany

**Keywords:** Alzheimer’s disease, elderly, hippocampal parcellation, metabolic covariance, networks

## Abstract

**INTRODUCTION:**

Hippocampal local and network dysfunction is the hallmark of Alzheimer’s disease (AD).

**METHODS:**

We characterized the spatial patterns of hippocampus differentiation based on brain co-metabolism in healthy elderly participants and demonstrated their relevance to study local metabolic changes and associated dysfunction in pathological aging.

**RESULTS:**

The hippocampus can be differentiated into anterior/posterior and dorsal cornu ammonis (CA)/ventral (subiculum) subregions. While anterior/posterior CA show co-metabolism with different regions of the subcortical limbic networks, the anterior/posterior subiculum are parts of cortical networks supporting object-centered memory and higher cognitive demands, respectively. Both networks show relationships with the spatial patterns of gene expression pertaining to cell energy metabolism and AD’s process. Finally, while local metabolism is generally lower in posterior regions, the anterior–posterior imbalance is maximal in late mild cognitive impairment with the anterior subiculum being relatively preserved.

**DISCUSSION:**

Future studies should consider bidimensional hippocampal differentiation and in particular the posterior subicular region to better understand pathological aging.

## 1 | NARRATIVE

### 1.1 | Contextual background

What are subregional differences in the hippocampal metabolic pattern in the aging population? The hippocampus is a core region in the study and understanding of brain diseases, in particular, in Alzheimer’s disease (AD)-related pathology.^1^ Previous studies have evidenced that the accumulation of tau (and to a lesser extend amyloid beta [A*β*]) pathologies in the hippocampus is associated with metabolic and structural changes of the hippocampus itself, but also of other brain regions in healthy aging and dementia populations,^2,3^ hence suggesting a role of hippocampal networks in AD pathology. Understanding the neurobiological properties of the hippocampus is therefore a crucial step to understand pathophysiological processes in AD. In that perspective, it has been pointed out that the disruption of its white matter fiber bundles, the spatiotemporal patterns of alteration of metabolism in spatially remote brain regions, and the pattern of macrostructural atrophy in the hippocampus in the early stages of AD are related events occurring as the neuropathology of AD spreads.^4–8^ Accordingly, a better understanding of AD neuropathology requires us to consider the hippocampus in interaction with other brain regions.

At the microbiological level, many studies have discussed the role of oxidative stress, mitochondrial dysfunction, and cellular calcium dysregulation in AD pathophysiology.^9–11^ Although at the microbiological level, several aspects of the cascade of cellular dysfunction remain unclear, at the macrobiological level, these dysfunctions lead to altered glucose metabolism in brain gray matter (GM) tissue. In particular, posterior cortical hypometabolism is a key early feature of the AD clinical picture. In contrast, hippocampal hypometabolism is less evident in patients, especially at the early stage of mild cognitive impairment (MCI), at which alterations may potentially take the form of hypermetabolism,^12^ as observed in other diseases.^13^ These complex metabolism patterns complicate the study of the relationships between hippocampal metabolism and other brain alterations. To delineate the relationships between hippocampal metabolism and whole-brain metabolism in healthy aging in the first place, the covariance between hippocampal metabolism and whole-brain metabolism should be examined. However, this approach is challenged by the fact that the hippocampus is not a uniform, homogeneous region with regard to several neurobiological features. In other words, different subregions should be considered separately within the hippocampus.

Which subregions should be considered for studying metabolic patterns? Cytoarchitecture studies,^1,14,15^ and structural^16,17^ and functional^18–21^ neuroimaging studies of the hippocampus resulted in divergent parcellation maps. In other words, different organizational dimensions can be seen within the hippocampus depending on which neurobiological aspect is probed. In particular, based on large-scale functional integration supporting behavioral systems in humans, the hippocampus can be subdivided along the anterior–posterior axis, while looking at local microstructural features (such as cytoarchitecture) typically reveals different subfields arranged along a medial–lateral and ventro–dorsal axis (such as cornu ammonis [CA]1, CA2, CA3, CA4, the subiculum, and the dentate gyrus).^22^ Thus, heterogeneity within the hippocampus has been evidenced based on local microstructure, connectivity profiles, and behavioral engagement, but how this heterogeneity influences metabolic patterns remains an open question.

### 1.2 | Results, implications, and limitations of the study

This study aimed to identify hippocampal subregions that each exhibit a specific spatial pattern of glucose metabolism in the brain, as representing hippocampal brain “metabolic networks.” To do so, we used a data-driven approach in which hippocampal voxels are clustered based on their individual profile of covariance with other brain regions across a sample of healthy elderly participants. By doing so, we identified five subregions that subdivide the hippocampus both along the medial–lateral/ventro–dorsal axis and along the anterior–posterior axis. These regions roughly correspond to the anterior CA field, the posterior CA field, an intermediate CA region, the anterior subiculum, and the posterior subiculum. We could then show that these subregions are relevant for studying local metabolism within the hippocampus as a specific pattern of significant differences could be seen in which glucose metabolism is greater in the subiculum than in the CA field and additionally, glucose metabolism is greater in anterior subregions than in posterior subregions in the healthy elderly sample. This anterior–posterior metabolic difference may be related to the increased and decreased functional connectivity of, respectively, the anterior medial and posterior medial temporal network, that have been observed with age in the healthy elderly population.^23^ Our results hence add to the growing literature highlighting hippocampal anterior–posterior differentiation and here suggest that it is crucial to consider this differentiation when examining glucose metabolism.

To further investigate whether this anterior–posterior differentiation is truly relevant in the study of AD, we looked at the individual difference (delta) between hippocampal anterior and posterior metabolism in a range of participants at different stages of the disease (*n* = 846), including healthy older with normal cognition, patients with early MCI, patients with late MCI (who are differentiated from the early MCI based on more severe memory deficit^24^), and patients with AD. By doing so, we observed that the delta (difference between anterior and posterior hippocampal metabolism) is maximal at the late MCI stage, that is, at the stage at which cognitive deficit becomes evocative of AD. Furthermore, group comparison of local metabolism for each region between the groups suggests a relative preservation of the anterior subiculum in late MCI compared to early MCI.

How does this anterior–posterior imbalance relate to behavior? One way to address this question would be to identify how the individual anterior–posterior delta relates to a range of neuropsychological symptoms spanning different cognitive functions, emotion, socio-affective processing, and neuropsychiatric symptoms. However, such data were not available for large clinical samples. Therefore, to explore this question in a system neuroscience approach, we examined the pattern of brain regions co-varying at the metabolism level with each hippocampal subregion. In other words, we computed the metabolic network of each hippocampal subregion. We then characterized the behavioral and neurotransmitter systems in which these networks are engaged.

This systemic characterization showed that CA regions are integrated in the subcortical limbic network, showing association with the amygdala and emotional processes for the anterior CA, but with an association with the dorsal attention, thalamus, and the noradrenergic system for the posterior CA. These associations seem to be related to the fornix path, which plays a significant role in interconnecting the hippocampal formation with the subcortical structures. For example, the post-commissural columns part of the fornix principally connects the hippocampal formation with the thalamus and hypothalamic nuclei.^25^ In contrast, subicular regions show mainly a cortical pattern of metabolic covariance. Despite the shared cytoarchitecture pattern in the anterior-subiculum (red) and posterior-subiculum (blue) these two regions show relatively different metabolic networks. In line with the literature,^18^ our results showed that the anterior subiculum is associated to a cortical network supporting “bottom-up” cognition for object identification and language comprehension while the posterior subiculum is integrated in several brain networks supporting higher cognitive demands such as memory retrieval, working memory, and spatial cognition. Accordingly, a pathological metabolic imbalance between anterior and posterior subregions would lead to a more pronounced cognitive deficit: while emotional and object recognition processes supported by anterior hippocampal networks would be more preserved, high level cognitive processes required for memory retrieval and spatial cognition supported by posterior hippocampal networks would be affected at the late MCI stage. It is thus crucial to consider hippocampal anterior–posterior differentiation to better understand behavioral phenotypes in patients with AD and likely in many other pathologies affecting the hippocampus.

After examining how subhippocampal metabolic networks relate to systems supporting behavioral phenotype, we explore how these networks relate to the AD pathophysiology by examining the spatial correspondence between maps of gene expression related to this latter and the brain metabolic covariance map of each metabolic subregion. We found that the spatial pattern of both the anterior and posterior subiculum were positively associated with the spatial pattern of gene expression pertaining to calcium-mediating signaling, respiratory electron transport chain, mitochondrial adenosine triphosphate (ATP) synthesis, cellular metabolic process, and upstream/downstream AD regulator genes. In contrast, the maps of the CA subregions did not significantly correlate with the spatial pattern of these genes’ expression. Therefore, our exploratory analyses suggest that the cellular dysfunction in the AD pathology might primarily be associated with subicular network dysfunction at the brain macroscale.

However, our results do not allow any insight on how the microbiological cascade relates to hippocampal glucose metabolism anterior–posterior imbalance (and possible decoupling) in AD. Recent studies suggest that the fatal disruption of cellular equilibrium is related to the join effects and interaction between mitochondrial dysfunction, altered calcium homeostasis, oxidative stress, A*β* plaques, and tau pathology.^26–30^ Yet, some studies suggest that the posterior hippocampal subregions are more affected by A*β*/tau pathologies than is the rostral hippocampus.^31,32^ Accordingly, the interaction between, on one hand, mitochondrial dysfunction, altered calcium homeostasis, and oxidative stress particularly expressed within the subicular network and, on the other hand, A*β* and tau pathology particularly affecting the posterior hippocampal network could result in a particularly detrimental pattern of alterations in the posterior hippocampal network leading, in turn, to typical episodic memory and spatial cognition impairments.

In sum, in this work, we delineated hippocampal subregions and their associated metabolic networks in the healthy older populations. We showed that, already in healthy older participants, glucose metabolism varies, on one hand, between the ventral–medial subregion corresponding to the subiculum and the dorsal–lateral subregion corresponding to the CA, but also, on the other and, within these fields, between anterior and posterior subregions. We assume that this anterior–posterior difference may be related to different changes in connectivity across aging between the anterior and the posterior medial temporal lobe networks in which the anterior one tends to increase while the posterior one tends to decrease.^23^ Nevertheless, specific studies using a longitudinal design would be needed to test the association between changes in the local metabolism of hippocampal anterior and posterior subregions and changes in their functional connectivity across aging.

We then further showed that anterior–posterior imbalance is maximal in patients with late MCI with relatively pronounced episodic memory deficit. Our characterization of the anterior and posterior hippocampal metabolic networks supports this finding by suggesting that a relative preservation of anterior hippocampal function with an alteration of the posterior hippocampal function would result in deficits in tasks with high cognitive demands while more bottom-up processes, needed for object recognition and language basic comprehension would be relatively preserved. Nevertheless, this hypothesis could be tested in the future by capitalizing on a deep behavioral phenotyping in a range of healthy older populations and patients to examine how the anterior–posterior imbalance at the individual level relates to individual cognitive and socio-affective functioning.

Based on the spatial correspondence between the pattern of subicular metabolic covariance across the brain and the patterns of gene expression engaged in the cell respiratory AD pathophysiological processes, we hypothesize that the subicular networks are particularly vulnerable to cellular dysfunction assumed for AD. To account for the greater decrease of metabolism in posterior regions, we speculate an interplay with tau and A*β* pathology that would particularly affect the posterior subicular network. This hypothesis should nevertheless be robustly tested using longitudinal design and A*β* /tau positron emission tomography (PET) markers to delineate the cascade of pathological events within the different hippocampal subnetworks across time.

## 2 | CONSOLIDATED RESULTS AND STUDY DESIGN

**FIGURE 1:**
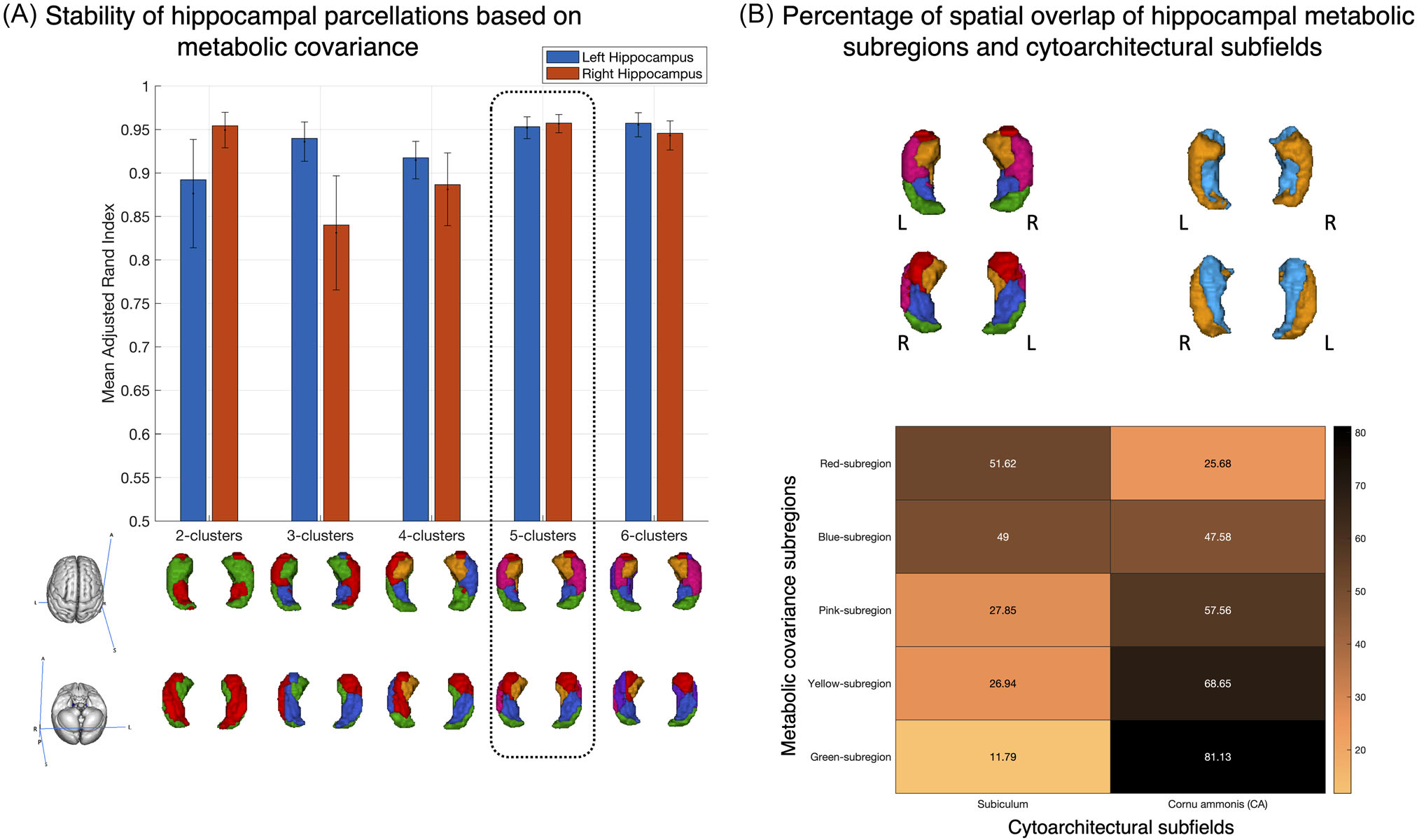
Stability and consistency of hippocampal parcellations based on metabolic covariance. A, Stable metabolic differentiation patterns were found for the right and left hippocampus for five-cluster solution estimated with split-half cross-validation using mean adjusted rand index. 3D view of different levels of parcellation (k = 2–6) from superior and inferior view are illustrated. B, Percentage of spatial overlap of hippocampal metabolic subregions and cytoarchitectural subfields are calculated and the subregions were named as follow: anterior-subiculum (red), posterior-subiculum (blue), intermediate-subregion (pink), anterior-CA (yellow), and posterior-CA (green). CA, cornu ammonis.

**FIGURE 2:**
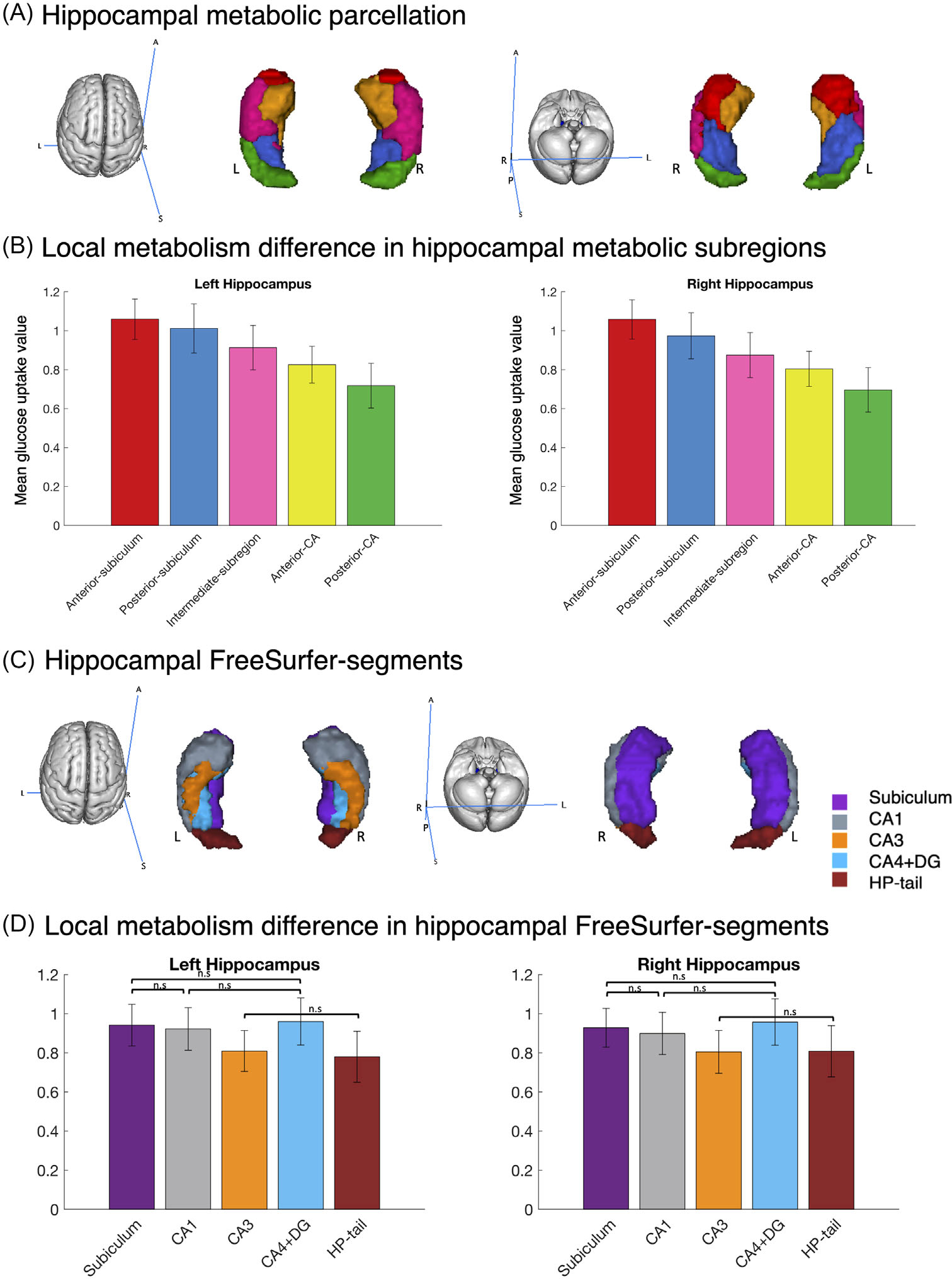
Local metabolism difference in hippocampal metabolic subregions and in FreeSurfer segment in a subsample of healthy elderly. A, Hippocampal metabolic parcellation. B, Local metabolism difference between hippocampal metabolic subregions in a subsample of healthy older subjects with high image resolution (≤ 2 mm). The difference between the mean normalized glucose uptake values of all hippocampal metabolic subregions across healthy older subjects was evaluated. Analysis of variance showed significant differences between metabolic subregions in their mean normalized glucose uptake value. For both hippocampi, a post hoc analysis revealed significant differences (corrected for multiple comparisons, *P*-value_FWE_ < 0.05) between all pairs of subregions. Overall, the profile of local metabolism in the metabolic networks-based parcellation strikingly shows a clear differentiation between subicular and CA subregions in which metabolism is typically higher in the former than the latter while the intermediate subregion (pink) stands between the two. C, Hippocampal subfield segmentation using automated pipeline in FreeSurfer v.6.0.^50^ D, Local metabolism difference in hippocampal segments, a subsample of healthy older subjects with high image resolution (≤ 2 mm). The hippocampal FreeSurfer segments show less sensitivity to local differences in glucose metabolism with many non-significant differences between segments. Thus, our results suggest that metabolic subregions accounting for an anterior–posterior differentiation offers a more sensitive model than FreeSurfer segments to study hippocampal subregional local metabolism. CA, cornu ammonis; DG, dentate gyrus; HP-tail, hippocampal tail; n.s, not significant.

The identification of metabolic subregions and their metabolic networks is an essential step to study and better understand the pathological processes in AD. To address this first objective in the current study, we performed a hippocampus parcellation based on a glucose metabolism profile computed from 18-fluorodeoxyglucose (^18^FDG) PET in a sample of 99 healthy elderly participants with high image resolution (≤ 2 mm) from the Alzheimer’s Disease Neuroimaging Initiative (ADNI) database. To do so, we first computed the glucose co-metabolism profile across the brain for each hippocampal voxel (i.e., the brain covariance pattern for each hippocampal voxel). We then clustered the hippocampal voxels based on the (dis)similarity of their whole-brain co-metabolism profile. We hence identified five subregions reflecting co-metabolism differentiation along both the anterior–posterior and ventro–dorsal/medio–lateral axes of the hippocampus (Figure 1). Based on the percentage of overlap between each yielded subregion and the CA versus subiculum fields defined by cytoarchitecture, the subregions were named as follow: anterior-subiculum (red), posterior-subiculum (blue), intermediate-subregion (pink), anterior-CA (yellow), and posterior-CA (green; Figure 1B and Figure 2A).

An examination of the local metabolism difference in the yielded hippocampal metabolic subregions in those 99 healthy older subjects demonstrated their relevance by highlighting significant differences between the CA and subiculum (while the intermediate subregion [pink] stands between the two), but also within these fields between the anterior and posterior subregions (Figure 2B). In contrast, the hippocampal FreeSurfer segments (Figure 2C) typically based on anatomical information show less sensitivity to local differences in glucose metabolism with many non-significant differences between segments (Figure 2D). Thus, our results suggest that metabolic subregions accounting for an anterior–posterior differentiation offer a more sensitive model than FreeSurfer segments to study hippocampal subregional local metabolism.

**FIGURE 3:**
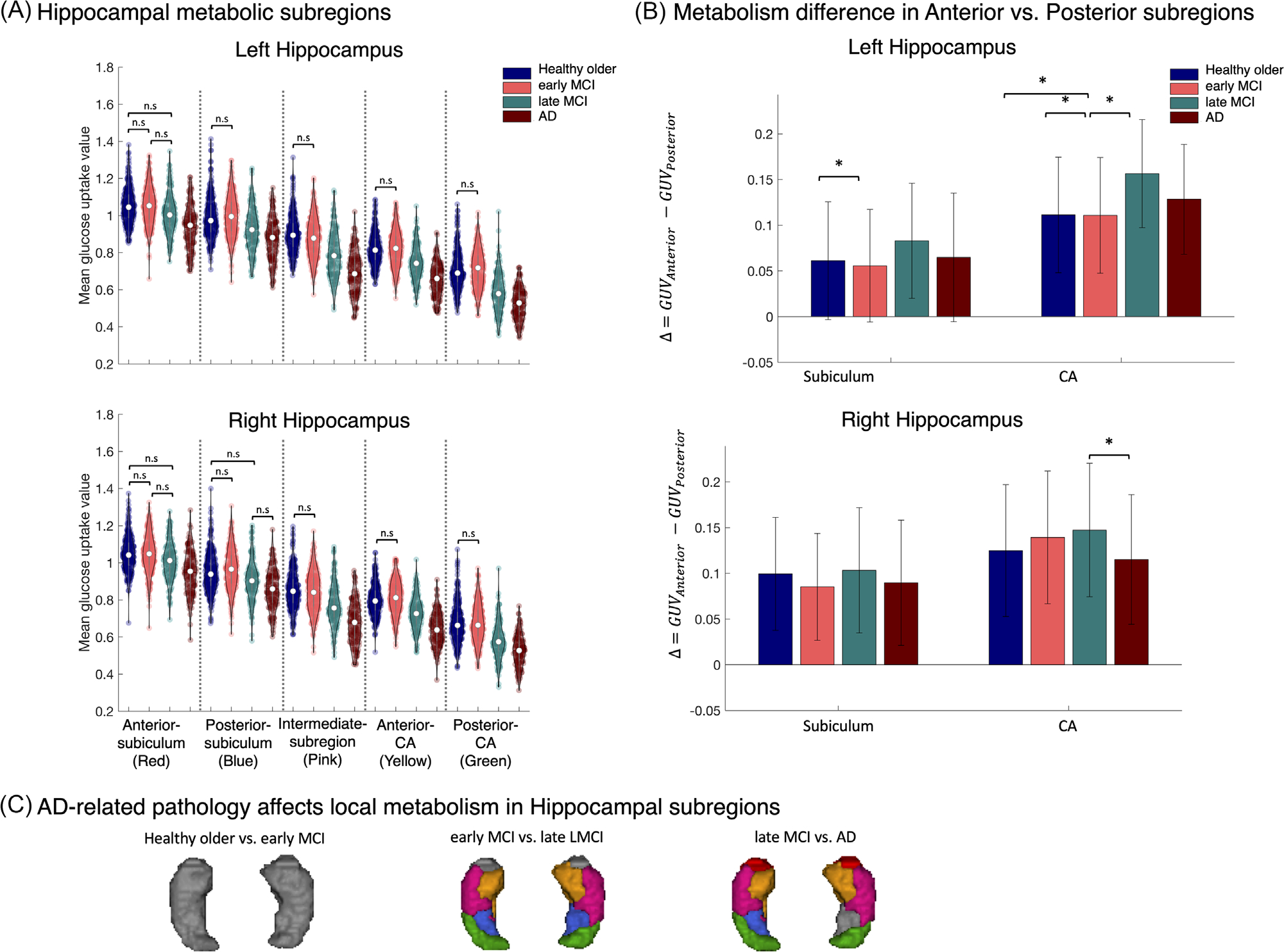
Alzheimer’s disease pathology’s effect on the local metabolism of hippocampal metabolic subregions; a comparison between groups being defined based on ADNI standard classification. A, The difference between mean normalized glucose uptake value of all metabolic subregions across all groups was evaluated by computing a two-way ANOVA with groups as one factor, hippocampal metabolic subregions as a second factor, and the mean normalized glucose uptake value as a dependent variable. Our results showed that there were significant main effects of both hippocampal metabolic subregions and groups. The ANOVA also yielded significant interaction effect between hippocampal metabolic subregions and groups. All comparisons between groups were significant according to a post hoc analysis while corrected for multiple comparisons (*P*-value_*FWE*_ < 0.05) except healthy older subjects versus early MCI comparison and for early MCI versus late MCI comparison in anterior-subiculum (red). B, Exploring the relevance of the anterior–posterior differentiation in the study of AD, we looked at the individual difference (delta) between hippocampal anterior and posterior metabolism in a range of participants at different stage of the disease, including healthy older participants and patients with early MCI, late MCI, and AD. We observed that the delta (difference between anterior and posterior hippocampal metabolism) is maximal at the late MCI stage, that is, at the stage at which cognitive deficit become evocative of AD. C, Group comparison of local metabolism for each region across all groups suggests a relative preservation of the anterior subiculum in late MCI compared to early MCI. AD, Alzheimer’s disease; ADNI, Alzheimer’s Disease Neuroimaging Initiative; ANOVA, analysis of variance; MCI, mild cognitive impairment.

**FIGURE 4:**
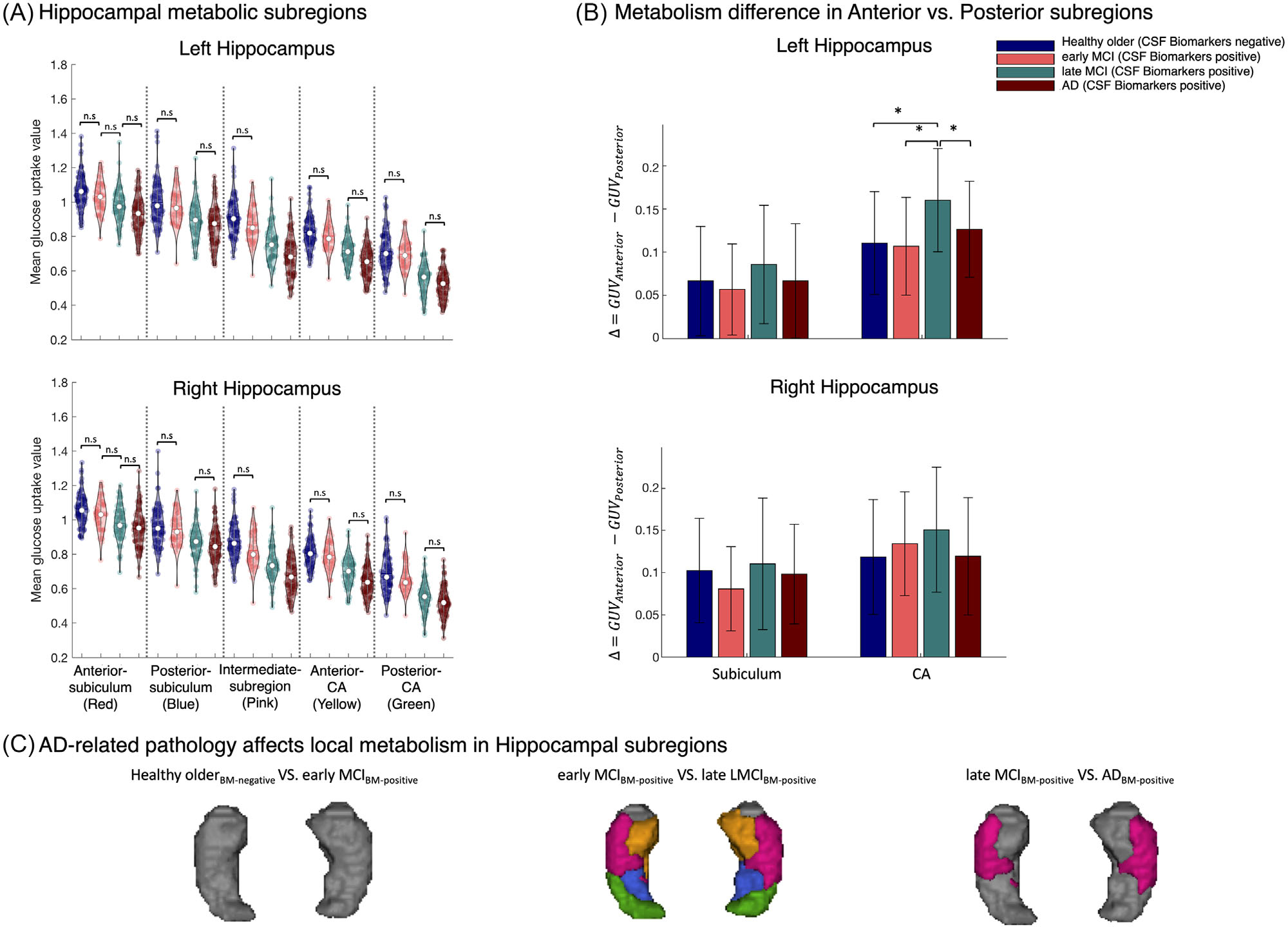
Alzheimer’s disease pathology’s effect on the local metabolism of hippocampal metabolic subregions; a comparison between groups being defined based on CSF AD biomarkers. A, The difference between mean normalized glucose uptake value of all hippocampal metabolic subregions across pathologically defined groups based on CSF AD biomarkers as the indicators of AD pathology (see Table S2 in supporting information) was evaluated by computing a two-way ANOVA with pathological groups as one factor, hippocampal metabolic subregions as a second factor, and the mean normalized glucose uptake value as a dependent variable. Our results showed that there were significant main effects of both hippocampal metabolic subregions and pathological groups. All comparisons between pathological groups were significant according to a post hoc analysis while corrected for multiple comparisons (*P*-value_*FWE*_ < 0.05) except early MCI (BM positive) versus healthy older subjects (BM negative), late MCI (BM positive) versus early MCI (BM positive) and AD (BM positive) versus late MCI (BM positive) comparisons. In bilateral hippocampi, there was no significant difference between healthy older subjects (BM negative and early MCI (BM positive). For late MCI (BM positive) versus early MCI (BM positive) comparison in anterior-subiculum (red), the level of metabolic glucose uptake value was preserved in late MCI (BM positive) patients and there was no significant difference. AD (BM positive) versus late MCI (BM positive) comparisons showed no further alteration in CA and subiculum subregions. B, Exploring the relevance of the anterior–posterior differentiation in the study of AD, while focusing specifically on grouping subjects in each diagnostic group based on CSF AD biomarkers as indicator of AD pathology. We observed a similar pattern to Figure 3B where at the late MCI stage the delta (difference between anterior and posterior hippocampal metabolism) is maximal. C, Here, we also observed a similar pattern to Figure 3C except comparing patients with AD dementia to late MCI for which no further alteration in CA and subiculum subregions appear significant. AD, Alzheimer’s disease; ANOVA, analysis of variance; BM, biomarkers; CA, cornu ammonis; CSF, cerebrospinal fluid; MCI, mild cognitive impairment.

Accordingly, we then examined how the local metabolism in these subregions is influenced by AD pathology in a cohort of ADNI participants (*n* = 846) (Table S1 in supporting information) using a two-way analysis of variance (ANOVA). Overall, a general decreasing trend in metabolism from early MCI to dementia groups was observed. However, no significant differences between healthy older participants and early MCI patients were observed. Actually, visually, early MCI patients seem to show increased local metabolism, in particular in the posterior subiculum (compared to healthy older participants), although this difference was not significant as several healthy older participants also appear as outliers with high local metabolism (Figure 3A). In contrast, comparing local metabolism between late MCI and early MCI reveals decreased metabolism in most hippocampal subregions in late MCI, although the anterior-subiculum (red) appeared relatively preserved. As our region-wise group comparison suggested different alterations of the anterior versus posterior regions across patient groups, we examined anterior–posterior difference at the individual level (individual delta value) and observed that the discrepancy is maximal in the late MCI patients (Figure 3B), that is, MCI patients with relatively more pronounced episodic memory deficits (see ADNI classification^24^). We repeated this analysis with patient diagnostic groups further selected grouped based on cerebrospinal fluid (CSF) AD biomarkers (novel Elecsys CSF immunoassays including the 42 amino acid-long A*β* peptide [A*β*(1–42)], the total tau protein [t-tau], and phosphorylated tau [p-tau]) to define healthy older biomarker (BM)-negative subjects, early/late MCI, and AD BM-positive subjects (*n* = 290; Table S2 in supporting information).^33,34^ Although the number of subjects was reduced (compared to the analysis using ADNI standard classification), we observed a similar pattern except comparing patients with AD dementia to late MCI in which no further alteration in CA and subiculum subregions appeared significant (Figure 4).

**FIGURE 5:**
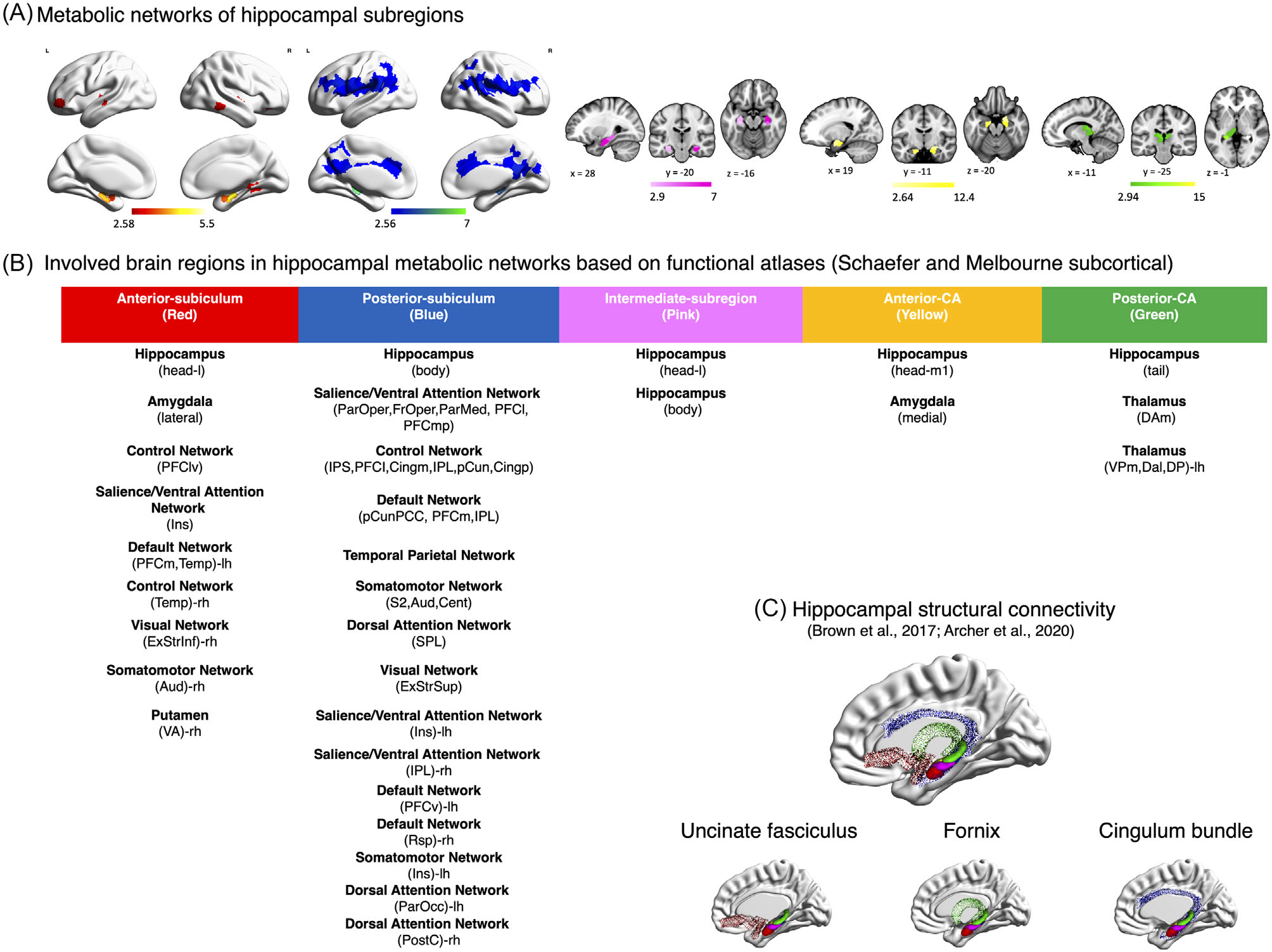
Main effect patterns of metabolic covariance of each hippocampal subregion in a subsample of healthy elderly participants with high image resolution. A, All patterns were corrected for multiple comparisons at the significance level of *P*-value_*FDR*_ < 0.05. B, List of involved brain regions in each hippocampal metabolic network based on functional atlases (Schaefer and Melbourne subcortical). C, Medial temporal lobe structural connectivity from diffusion magnetic resonance imaging studies.^58,59^ The main tracts are represented with transparent colors together with the hippocampal metabolic subregions (shown in plain colors).

To better understand the neurobiological correlates of each subregion in the healthy population, we characterized their metabolic networks using a general linear model that quantifies, for each subregion, its association to each brain region with regard to covariance in glucose metabolism. We here only report the region that shows significant associations (Figure 5A). Despite the shared cytoarchitecture pattern in the anterior-subiculum (red) and posterior-subiculum (blue) these two regions show relatively different metabolic networks. Overall, anterior-subiculum (red) show primarily metabolic covariance with the prefrontal and temporal regions as well as the insula, the extrastriate inferior cortex, the lateral amygdala, and putamen suggesting a network corresponding to input information from anterior regions into the hippocampal head, partially through the uncinate fasciculus (see Figure 5C). In contrast, the posterior-subiculum (blue) showed the widest brain metabolic covariance pattern, overlapping with most of the seven Yeo functional brain networks and including the intraparietal sulcus, the superior parietal cortex, inferior frontal regions, but also the cingulate cortex (which is anatomically connected to the medial temporal lobe through the cingulum bundle, see Figure 5C). The intermediate-subregion (pink) appears as a transitional subregion in this differentiation showing only local covariance (within the hippocampus). Finally, the Aanterior-CA (yellow) relates to the amygdala while posterior-CA (green) relates to the thalamus (which is anatomically connected to the hippocampus through the fornix, see Figure 5C).

**FIGURE 6:**
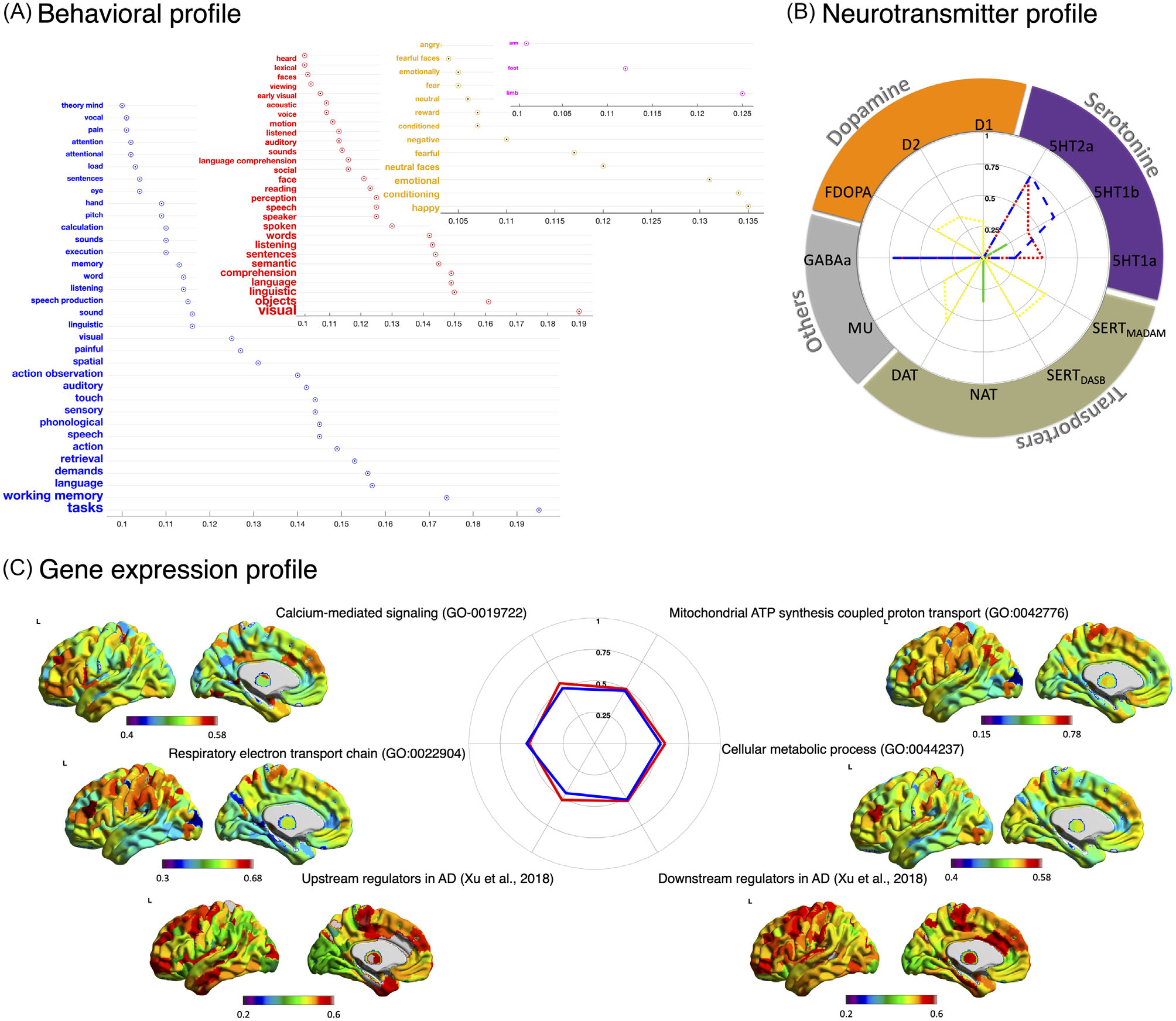
Behavioral, molecular, and AD-related genetic characterization of hippocampal metabolic subregions’ networks in a subsample of healthy elderly participants. A, The line plots summarize the topics most associated with the metabolic covariance pattern of anterior-subiculum (red), posterior-subiculum (blue), anterior-CA (yellow), and intermediate-subregion (pink). Larger words are more associated with networks (the associated value is based on the calculated *r*-value in NeuroSynth representing the spatial correlation). B, Topographical relationship between a whole-brain metabolic network of bilateral hippocampal metabolic subregions and several neurotransmitter maps explored to assess for highly expressed receptors/transporters in each metabolic network relative to the whole brain using the JuSpace toolbox.^52^ The radial values are *r*-values representing the spatial correlations. We used the dotted line to make overlap patterns clear. C, The spatial maps of AD-related gene expression that are disrupted in enriched pathways in AD hippocampi^53^ were calculated using *post mortem* gene expression data from the AHBA as a reference.^54^ After that, the topographical relationship between a whole-brain metabolic network of hippocampal metabolic subregions and those spatial maps were explored to assess for highly expressed genes in each metabolic network relative to the whole brain. The radial values are *r*-values representing the spatial correlations. AD, Alzheimer’s disease; AHBA, Allen Human Brain Atlas; CA, cornu ammonis.

To better understand how the metabolic covariance pattern of each subregion relates to neurobiological and behavioral systems, as well as to AD pathophysiology, we compared the unthresholded spatial map of metabolic covariance of each subregion to (1) meta-analytic maps of activations for behavioral functions, (2) neurotransmitter system maps, and (3) AD-related gene expression maps. Of note, the use of the unthresholded map allow us to retain the whole spatial pattern of variation across the brain (rather than focusing on a few regions that are significant) and to compare this pattern to patterns of different neurobiological features. By doing so, we found that the anterior-subiculum (red) brain pattern was related to object recognition while the posterior-subiculum (blue) was related to higher cognitive function such as memory retrieval, working memory, and spatial cognition (Figure 6A). Both subicular brain patterns (anterior-subiculum [red] and posterior-subiculum [blue]) were significantly similar to the brain pattern of genes related to AD pathophysiology (Figure 6C). Furthermore, both subicular patterns significantly overlap with the serotonergic system and the GABAA receptor distribution (Figure 6B). In contrast, the anterior-CA (yellow) brain pattern was related to the emotional system (Figure 6A). The anterior- and posterior-CA brain metabolic patterns did not show significant spatial overlap with the investigated gene expression patterns, but were associated with the dopaminergic and serotonergic systems (Figure 6B). The posterior-CA (green) pattern additionally showed significant association with the noradrenergic system (Figure 6B). Finally, the intermediate-subregion (pink) brain pattern was only associated with body-part terms such as limb, foot, and arm and did not show significant overlap with neurotransmitter and gene expression systems.

## 3 | DETAILED METHODS AND RESULTS

### 3.1 | Methods

#### 3.1.1 | Imaging dataset

The structural magnetic resonance imaging (MRI) and ^18^FDG-PET scans of 846 elderly healthy participants, MCI, and dementia participants including early/late MCI and AD were obtained from openly accessible neuroimaging ADNI cohort (visit http://adni.loni.usc.edu/ for information about scanning parameters). The demographic data are reported in Table S1. These subjects were also further categorized into four groups based on CSF AD biomarkers using the novel Elecsys 42 amino acid-long A*β* peptide (A*β*_(1–42)_), t-tau, and p-tau). CSF immunoassays provide a continuous, quantitative measure of AD-related proteins. We used these three core CSF biomarkers currently being used for AD diagnostics to define healthy older BM-negative subjects, early/late MCI, and AD BM-positive subjects.^33,34^ We used pre-established cut-offs for CSF AD biomarkers to define BM-negative/positive subjects.^33,34^ The cut-off for A*β*(1–42) CSF AD biomarkers measured by using novel Elecsys CSF immunoassays and optimized for concordance with amyloid-PET visual read were defined as 977 pg/mL (A*β*[1–42]).^33^ The cut-offs for p-tau and t-tau CSF AD biomarkers have been previously optimized for identification of AD patients versus normal controls in the ADNI populations by a sensitivity analysis.^34^ The identified cut-offs were 24 pg/mL (p-tau) and 266 pg/mL (t-tau) in ADNI.^34^ In Table S2, we reported global cognition as well as information on CSF AD biomarkers. We downloaded the preprocessed ^18^FDG-PET images labeled as “CO-REGISTERED, AVERAGED.” The details of preprocessed ^18^FDG-PET image data available in ADNI can be found at http://adni.loni.usc.edu/methods/pet-analysis. The analysis of these data was approved by the ethical committee of the Heinrich Heine University Düsseldorf. All ^18^FDG-PET and structural MRI images used in this research were preprocessed using the pet-volume pipeline and the t1-volume pipeline of Clinica, respectively.^35,36^ The details of Clinica pipelines and preprocessing steps are provided in the following sections.

#### 3.1.2 | Structural MRI data preprocessing

All structural MRI images were preprocessed by using the t1-volume pipeline of Clinica.^35,36^ This pipeline is a wrapper of the segmentation and normalization to the Montreal Neurological Institute (MNI) space^37^ routines implemented in SPM12. First, the MRI images were bias-field corrected, segmented into GM, white matter, and CSF, and spatially normalized using the Unified Segmentation procedure.^38^ Next, DARTEL^39^ used the subjects’ tissue probability maps on the native space obtained at the previous step to create a group template. Then, the DARTEL to MNI registration approach introduced in Ashburner^39^ was applied to modulate the GM segments with the nonlinear transformations from subject space into the MNI space and subsequently smoothed with an isotropic Gaussian kernel (full width half-maximum [FWHM] = 8 mm).

#### 3.1.3 | Quality assessment of the ^18^FDG-PET data for use in the hippocampal parcellation

The ADNI dataset is characterized by a high variability regarding image resolution (voxel size of the native ^18^FDG-PET images; Table S3 in supporting information). The image resolution in ^18^FDG-PET is typically smaller than 4 mm. However, for specific structures like the hippocampus, higher image resolution may be needed. Previous parcellation studies of the hippocampus were performed either in structural MRI data or in functional MRI data,^17–19,40^ which typically had an image resolution ≤ 2 mm. Accordingly, we set 2 mm as the acceptable image resolution for ^18^FDG-PET data in this study to define broad subregions within the hippocampus. The ^18^FDG-PET data of only 99 subjects from the sample of 266 cognitively normal participants passed this criterion and were therefore used for hippocampal parcellation based on metabolic covariance profile.

#### 3.1.4 | ^18^FDG-PET data preprocessing

All ^18^FDG-PET scans used in this research were preprocessed using the pet-volume pipeline of Clinica.^35,36^ First, intra-subject registration of the ^18^FDG-PET image into the space of the subject’s structural MRI image was done using SPM12 toolbox. Thus, each subject’s coregistered image from the baseline PET scan is in the subject’s native space 170 × 256 × 256 voxel image grid, having 1.2 × 1 × 1 mm^3^ voxels. Then, the ^18^FDG-PET images were corrected for partial volume effects using the PETPVC toolbox.^41^ In the PETPVC toolbox eight core partial volume correction (PVC) techniques are available. These core methods can be combined to create a total of 22 different PVC techniques. CLINICA developers used region-based voxel-wise correction approach. The corrected ^18^FDG-PET images were then spatially normalized into MNI space using the DARTEL deformation model of SPM12 (each subject’s corrected ^18^FDG-PET scan was normalized into a standard 121 × 145 × 121 voxel image grid, having 1.5 mm cubic voxels), and intensity normalized using the average glucose uptake value in a reference region (the whole pons). To prevent generating partial volume effect by smoothing data for denoising, only the GM segment of ^18^FDG-PET images was smoothed with an isotropic Gaussian kernel (FWHM = 8 mm). Thus, each subject’s GM segment of ^18^FDG-PET data was extracted by multiplying each subject’s preprocessed ^18^FDG-PET data with corresponding GM segment and smoothed. Finally, each subject’s smoothed GM segment of ^18^FDG-PET data was parcellated using a combination of the Schaefer atlas for 400 cortical regions^42^ and the Melbourne subcortex atlas for 54 subcortical regions.^43^ Because the subcortical atlas overlaps in some voxels with the cortical atlas, these voxels were set to zero (background) in the cortical atlas. This was done to avoid artificial correlation between metabolic uptake value of regions due to that overlap.

#### 3.1.5 | Metabolic covariance computation

Right and left human hippocampal volume of interest masks were created by combining cytoarchitecture maps available in the SPM Anatomy Toolbox 2.0,^44^ and the macro anatomically defined Harvard-Oxford Structural Probability Atlas (http://neuro.imm.dtu.dk/wiki/Harvard-Oxford_Atlas).^45^ The total number of voxels in a 2 mm × 2 mm × 2 mm space in the right hippocampus was 865 (6920 mm^3^) and that of the left hippocampus was 831 (6648 mm^3^) voxels. Hippocampal metabolic covariance was computed for the hippocampal voxels by correlating the hippocampal voxels with 454 brain parcels across the subsample of healthy older subjects with high image resolution (≤ 2 mm) using *z* transformed Pearson correlation. To ensure stability, a boostrapping approach for subjects^46^ was used by generating 500 bootstrap samples from which metabolic covariance matrix were computed.

#### 3.1.6 | Metabolic covariance-based parcellation

To identify hippocampal subregions based on the similarity/dissimilarity of their metabolic covariance pattern, we used k-means clustering implemented in MATLAB in line with previous studies.^18,47–49^ Based on the image resolution of ^18^FDG-PET data, we examined five levels of clustering, k = 2–6, and set the repetition number to 500, and the iteration number to 255, with random initialization in each iteration.^18^ A robust clustering solution was obtained by averaging the result of clustering across bootstrap samples. The final hippocampus parcellations were created by assigning each hippocampus voxels to its most frequent cluster’s label (i.e., by using the mode) across bootstrapping samples.^46^ The stability of the different levels of hippocampal partition (i.e., a number of the clusters) was evaluated by a split-half approach (10,000 splits) and by calculating the similarity between the two halves using the adjusted Rand Index with chance-level correction. Finally, we calculated the spatial overlap between the yielded stable hippocampal parcellation from metabolic covariance with the CA and subiculum fields defined based on Juelich cytoarchitecture probability atlas (Juelich-maxprob-thr25–2mm.nii) available in the FSL Toolbox. The percentage of spatial overlap for each hippocampal metabolic subregion was computed as the number of overlapping voxels in both the hippocampal metabolic subregion and the CA (or subiculum) field divided by the total number of the voxels in the hippocampal metabolic subregion and multiplied by 100.

#### 3.1.7 | Comparison between hippocampal metabolic parcellation and hippocampal FreeSurfer segments

First, we extracted hippocampal segments using FreeSurfer based on the preprocessed structural MRI images. To do so, individual normalized structural images were averaged for all subjects to create a group template used to generate hippocampal segments based on automated hippocampal subfield segmentation (FreeSurfer v.6.0).^50^ Twelve segments were obtained for each lateral hippocampus (Figure 2C). We focused on GM segments and defined five standard segments including the CA1, the CA3, a combination of the dentate gyrus and CA4 (C4 + DG), the hippocampal tail (HF-tail), and the subiculum as one merged segment (parasubiculum + presubiculum + subiculum). CA4 is adjacent to the dentate gyrus and not universally accepted as a distinct region. In line with common practices, we therefore here merged these two very small subfields into one segment.^15,51^ Then, we computed the mean normalized glucose uptake value for each segment for each subject in the healthy elderly and patient cohorts.

#### 3.1.8 | AD-related pathology effect on local metabolism in hippocampal metabolic subregions

To explore how AD pathology affects local metabolism differences across yielded hippocampal metabolic subregions in healthy older subjects, two-way ANOVA with post hoc comparison with correction for multiple testing were performed to identify statistical differences. We also repeated this analysis while focusing specifically on grouping subjects in each diagnostic group based on CSF AD biomarkers as indicator of AD pathology (see Table S2).

#### 3.1.9 | Metabolic networks of hippocampal metabolic subregions

We then determined the main metabolic network of each hippocampal subregion that have thus influenced the differentiation of hippocampal metabolic subregions, across the subsample of healthy older subjects with high image resolution (≤ 2 mm). We examined the main effect of metabolic covariance of each metabolic subregion across the whole-brain. To do so, we used the general linear model applied at the parcel level to investigate the linear relationship between the average glucose uptake value of the metabolic subregion of interest and every parcel in the whole-brain GM mask. The metabolic covariance pattern of each subregion across the whole-brain was computed with correction for false discovery rate (FDR) at the significance level of *P*-value < 0.05.

#### 3.1.10 | Behavioral characterization of hippocampal metabolic networks

To characterize hippocampal metabolic networks with regard to behavioral systems using the Neurosynth database (https://neurosynth.org/), we searched for whole-brain behavioral systems corresponding to metabolic networks by correlating the whole spatial pattern of metabolic network map with activation meta-analyses map and not for area-based engagement into specific behavioral function. The Neurosynth database comprises functional co-activation maps obtained from meta-analyses of task-functional MRI studies across 14,371 articles that are paired with sets of terms extracted from the text of these studies. The embedded cognitive decoding tool in Neurosynth contains > 1300 terms. So, we can compare each unthresholded T map of hippocampal metabolic network with the available collection of Neurosynth term-based meta-analysis maps using the embedded cognitive decoding tool. It computed Pearson correlations between our metabolic covariance maps and the term-based meta-analytic maps available in Neurosynth. Therefore, each term (e.g., “working memory”) had an associated value indicating the degree to which the brain metabolic map of a given hippocampal subregion resembles the activation map for that term. We included all correlations above 0.1 for terms pertaining to behavioral aspects to identify the broad pattern of behavioral systems overlapping with each hippocampal metabolic subregion’s network.

#### 3.1.11 | Molecular/neurotransmitter characterization of hippocampal metabolic networks

We also evaluated the topographical relationship between whole-brain hippocampal metabolic covariance and all neurotransmitter whole-brain maps available in the JuSpace toolbox.^52^ We investigated 12 different neurotransmitter maps including dopamine receptors (D1 and D2/D3 and F-DOPA [a reflection of presynaptic dopamine synthesis capacity]), serotonin receptors (5-HT1A, 5-HT1B, and 5-HT2A), transporters DAT (dopamine transporter), SERT (serotonin transporter with two different tracers DASB and MADAM), and NAT (noradrenaline/norepinephrine transporter) and GABAA and muopioid receptors (please see the article on the JuSpace toolbox^52^ for more information). To calculate the spatial correlation between each unthresholded T map of hippocampal metabolic network with all neurotransmitter maps by JuSpace, first, the 3D nifti file was loaded into the toolbox. Then the provided file and all the selected neurotransmitter maps were loaded into the atlas space to extract mean value per atlas region for each file. We used the default atlas (neuromorphometrics atlas from SPM12) excluding all white matter and CSF regions. Finally, spatial correlation was computed between the extracted values (while adjusting for spatial autocorrelation using the GM probability map TPM.nii from SPM12) by using Pearson correlation, and were then *z* transformed. Significant spatial association between brain metabolic patterns and each neurotransmitter map were examined by comparing the distribution of *z* transformed correlations against a null distribution using one-sample *t* tests while correcting for multiple comparisons using FDR correction (*P*-value_*FDR*_ < 0.05).

#### 3.1.12 | AD-related genetic characterization of hippocampal metabolic networks

Having established behavioral and neurotransmitter contextualization of our findings, we finally aimed to understand hippocampal metabolic networks’ association with gene expression profiles of AD-affected brain hippocampi. A recent systematic integrated analysis of expression profiles of AD-affected brain tissues including brain hippocampus (684 AD and 562 controls) reported a complete list of differentially expressed genes in an AD hippocampus (http://www.alzdata.org/file/HP_all_adj.txt) as well as hub genes of the transcriptomic network that upregulated and downregulated AD-related genes (Table S4 in supporting information).^53^ The biological pathways dysregulated in the hippocampus based on the identified differentially expressed genes related to the metabolic processes were significantly enriched, which indicated a dysregulation of energy metabolism in the hippocampus during the AD development.^53^ In this study, we extracted spatial gene expression maps related to enriched pathways (Gene Ontology [GO], biological process) of differentially expressed genes in the hippocampus including (GO:0044237~cellular metabolic process), (GO:0022904~respiratory electron transport chain), (GO:0042776~mitochondrial ATP synthesis coupled proton transport), (GO-0019722 ~ calcium-mediated signaling) from the processed *post mortem* gene expression maps available inAllen Human Brain Atlas (AHBA)^54^ (https://human.brain-map.org). In the following section, the processing detail of transcriptomic data provided by the AHBA is explained. To define the list of genes in each GO that are differentially expressed in the AD hippocampus, first the initial list of genes in each GO were extracted from http://geneontology.org/ database, restricted to human genes (Taxon: Homo sapiens). After that, intersection of each GO list with the list of differentially expressed genes in the AD hippocampus (http://www.alzdata.org/file/HP_all_adj. txt) defines the list of genes in each GO that are differentially expressed in the AD hippocampus. Furthermore, we investigated the topographical relationship of the hippocampal metabolic networks with the spatial distribution of gene expression in enriched pathways of differentially expressed genes in the hippocampus, as well as with the spatial distribution of hub genes of the transcriptomic network that upregulated and downregulated AD-related genes. To calculate the spatial correlation between each unthresholded T map of hippocampal metabolic covariance with all GO expression maps, we used Pearson correlation. These were adjusted for spatial autocorrelation and *z* transformed. Significant spatial associations between the subregion’s pattern of brain metabolic covariance and each GO expression map were examined by comparing the distribution of *z* transformed correlations to a null distribution using one-sample *t* tests while correcting for multiple comparisons using FDR correction (*P*-value_*FDR*_ < 0.05).

#### 3.1.13 | Transcriptomic data

Transcriptomic data provided by the AHBA (https://human.brain-map.org )^54^ was used to extract gene expression maps. Here, regional microarray expression data were processed with the abagen toolbox (version 0.1.3; https://github.com/rmarkello/abagen).^55^ Processing steps included probe-to-gene re-annotation, intensity-based data filtering, probe selection by mean, separating tissue samples into subcortical and cortical regions, normalization and aggregation within the Schaefer-400 cortical and the Melbourne subcortex 54 parcels in MNI-152 space and across six donors (1 female, age range: 24–57, mean age [standard deviation]: 42.50 [13.38]).

First, microarray probes were reannotated using data provided by Arnatkeviciute et al.;^55^ probes not matched to a valid Entrez ID were discarded. Next, probes were filtered based on their expression intensity relative to background noise,^56^ such that probes with intensity less than the background in ≥ 50% of samples across donors were discarded. When multiple probes indexed the expression of the same gene, we selected and used the probe with the most consistent pattern of regional variation across donors (i.e., differential stability^54^), calculated with:

$$\Delta_{S}(p)=\frac{1}{\left(\begin{array}{c} N\\ 2 \end{array}\right)}\sum_{i=1}^{N-1}\;\sum_{j=i+1}^{N}\;\rho \left[B_{i}(p),B_{j}(p)\right]$$

where ρ is Spearman’s rank correlation of the expression of a single probe, p, across regions in two donors $B_{i}$ and $B_{j}$, and N is the total number of donors. Here, regions correspond to the structural designations provided in the ontology from the AHBA.

The MNI coordinates of tissue samples were updated to those generated via non-linear registration using Advanced Normalization Tools (ANTs; https://github.com/chrisfilo/alleninf). Samples were assigned to brain regions by minimizing the Euclidean distance between the MNI coordinates of each sample and the nearest surface vertex. Samples in which the Euclidean distance to the nearest vertex was more than two standard deviations above the mean distance for all samples belonging to that donor were excluded. To reduce the potential for misassignment, sample-to-region matching was constrained by hemisphere and gross structural divisions (i.e., cortex, subcortex/brainstem, and cerebellum, such that, e.g., a sample in the left cortex could only be assigned to an atlas parcel in the left cortex^55^). If a brain region was not assigned a sample from any donor based on the above procedure, the tissue sample closest to the centroid of that parcel was identified independently for each donor. The average of these samples was taken across donors, weighted by the distance between the parcel centroid and the sample, to obtain an estimate of the parcellated expression values for the missing region. All tissue samples not assigned to a brain region in the provided atlas were discarded. Inter-subject variation was addressed by normalizing tissue sample expression values across genes using a robust sigmoid function:^57^

$$x_{\text{norm}}=\frac{1}{1+exp\left(-\frac{\left(x-\langle x\right\rangle }{IQR_{x}}\right)}$$

where ⟨x⟩ is the median and $IQR_{x}$ is the normalized interquartile range of the expression of a single tissue sample across genes. Normalized expression values were then rescaled to the unit interval:

$$x_{scaled}=\frac{x_{norm}\;-\;min\;\left(x_{norm}\right)}{max\;\left(x_{norm}\right)\;-\;min\;\left(x_{norm}\right)}$$

Gene expression values were then normalized across tissue samples using an identical procedure. Samples assigned to the same brain region were averaged separately for each donor and then across donors, yielding a regional expression matrix. Last, we excluded the right hemisphere regions due to the sparsity of samples in this hemisphere (data were only available for two donors) resulting in a large number of regions with no expression data. Finally, we obtained expression values for 15,631 unique genes at 400 cortical and 54 subcortical locations separately.

### 3.2 | Result

#### 3.2.1 | Stable hippocampal metabolic subregions

We evaluated the stability of each partition level by using split-half cross-validation. Overall, in both the left and right hippocampi, parcellation into five subregions were the most stable solution (Figure 1A).

Comparison of the yielded five-subregion partition to the subfields traditionally known from cytoarchitectural mapping suggested a CA field versus subiculum field with an anterior ventral (red) subregion resembling the anterior part of the subiculum and a posterior medial (blue) subregion that extends ventrally closely resembling the posterior part of the subiculum (Figure 1B). Quantitative spatial overlap with cytoarchitecture subfield further suggest two CA subregions: an anterior medial (yellow) subregion and a posterior lateral (green) subregion (Figure 1B). Accordingly, and for the reader’s convenience, in the next sections, the subregion will be labeled based on the correspondence with hippocampal subfields, their position on the longitudinal axis, and their color code: anterior-subiculum (red), posterior-subiculum (blue), intermediate-subregion (pink), anterior-CA (yellow) and posterior-CA (green; Figure 1B and Figure 2A).

#### 3.2.2 | Local metabolism in metabolic subregions versus FreeSurfer segments in the healthy older cohort

Both one-way ANOVA results in hippocampal metabolic subregions (Figure 2B) and FreeSurfer segments (Figure 2D) showed significant differences in their local metabolism. Hippocampal metabolic subregions’ results showed significant differences between the metabolic subregions in their mean normalized glucose uptake value (left: *F* [4494] = 153.24, *P*-value > 0.001; right: *F* [4494] = 169.23, *P*-value > 0.001; Figure 3A). Furthermore, a post hoc analysis revealed significant differences (corrected for multiple comparisons using family wise error rate [FWE], *P*-value_*FWE*_ < 0.05) between all pairs of metabolic subregions in their local metabolism for both hippocampi (Figure 2B). Hence, the profile of local metabolism in the metabolic networks-based parcellation strikingly shows a clear differentiation between subicular and CA subregions in which the metabolism was typically higher in the former than the latter while the intermediate subregion (pink) stands between the two. Furthermore, significant differences between anterior and posterior subregions were observed. FreeSurfer segment results also showed significant differences between the FreeSurfer segments in their local metabolism (left: *F* [4494] = 50.76, *P*-value > 0.001; right: *F* [4494] = 37.26, *P*-value > 0.001; Figure 2D). However, a post hoc analysis in FreeSurfer segments revealed many non-significant pair-wise differences such as between subiculum and CA1, between subiculum and CA4 + DG, between CA1 and CA4 + DG, and between CA3 and HP-tail (Figure 2D). Overall, these results suggest that metabolic subregions offer a more sensitive model than FreeSurfer segments to study hippocampal subregional local metabolism.

#### 3.2.3 | AD-related pathology affects local metabolism in hippocampal subregions

We examined the local metabolism differences in metabolic subregions across ADNI diagnosis groups including healthy older, early MCI, late MCI, and AD participants. Our results suggest that there might be a slight increase in local metabolism in early MCI (relative to healthy older subjects) in posterior subregions (Figure 3A). However, a statistical difference cannot be evidenced as several healthy older participants also show relatively higher local metabolism in posterior regions. Additionally, our parcellation map revealed that the global decrease between late and early MCI is not significant in the bilateral anterior-subiculum (red), a pattern that was also observed when focusing specifically on CSF AD BM-positive patients^33,34^ (Figure 4A). Moreover, our metabolic map specifically disentangling anterior from posterior subregions revealed that at the individual level, there is a differentiation between glucose uptake value of anterior–posterior regions in which the posterior has decreased metabolism compared to the anterior and that differentiation is maximal at the late MCI stage (Figure 3B and Figure 4B).

#### 3.2.4 | Local metabolism comparison in hippocampal subregions across groups while groups being defined based on ADNI standard classification

Exploring local metabolism difference in hippocampal metabolic subregions in healthy elderly participants and AD-related diagnosis groups showed a significant main effect of metabolic subregions (left: *F* [4, 4229] = 1617.88, *P*-value = 0; right: *F* [4, 4229] = 1805.16, *P*-value = 0). The main effect of groups was also significant (left: *F* [3, 4229] = 477.47, *P*-value = 0; right: *F* [3, 4229] = 381.67, *P*-value = 0) demonstrating highest/lowest metabolism rate for healthy older/dementia groups. The two-way ANOVA also revealed a significant interaction effect between metabolic subregions and diagnostic groups (left: *F* [12, 4229] = 7.65, *P*-value = 0; right: *F* [12, 4229] = 6.6, *P*-value = 0). In the bilateral hippocampus, all comparisons between groups in all subregions were significant according to a post hoc analysis corrected for multiple comparisons (*P*-value_*FWE*_ < 0.05) except for the healthy older subjects versus early MCI comparison in all subregions and for early MCI versus late MCI comparison in anterior-subiculum (red). In the right hippocampus, late MCI versus AD comparisons did not show significant differences except in posterior-subiculum (blue; Figure 3A and Figure 3C).

#### 3.2.5 | Local metabolism comparison in hippocampal subregions across groups while groups being defined based on CSF AD biomarkers

Here, we focused specifically on grouping subjects based on CSF AD biomarkers^33,34^ as indicators of AD pathology (see Table S2). To do so, we grouped participants in the healthy older diagnostic group for subjects who were BM-negative and in patient groups for subjects that were BM-positive. Then, we considered four different subgroups including healthy older subjects that were BM-negative (under the assumption that these subjects have no AD pathology at all), early MCI (BM-positive), late MCI (BM-positive), and AD (BM-positive; under the assumption that these subjects have AD pathology and symptomatic profile) to explore how local metabolism is affected by AD pathology. It should be noted that focusing on participants that are BM-positive/negative importantly reduces the number of participants in each group compared to analysis with ADNI classification (i.e., healthy older BM-negative [*n* = 80] versus healthy older [*n* = 266], early MCI BM-positive [*n* = 52] versus early MCI [*n* = 285], see Table S2).

When examining local metabolism, our results showed a significant main effect of metabolic subregions (left: *F* [4, 1449] = 606.94, *P*-value = 0; right: *F* [4, 1449] = 719.51, *P*-value = 0). The main effect of groups was also significant (left: *F* [3, 1449] = 245.03, *P*-value = 0; right: *F* [3, 1449] = 201.07, *P*-value = 0) demonstrating highest/lowest metabolism rate for healthy older/dementia groups. The two-way ANOVA also revealed a significant interaction effect between metabolic subregions and diagnostic groups, (left: *F* [12, 1449] = 3.26, *P*-value = 0.0001; right: *F* [12, 1449] = 3.04, *P*-value = 0.0003). In the bilateral hippocampus, all comparisons between groups in all subregions were significant according to a post hoc analysis corrected for multiple comparisons (*P*-value_FWE_ < 0.05) except for the healthy older (BM-negative) versus early MCI (BM-positive) where no region show significant differences. Furthermore, for early MCI (BM-positive) versus late MCI (BM-positive), the anterior-subiculum (red) did not show group effect. Finally in the late MCI (BM-positive) versus AD (BM-positive) comparisons, a significant difference was observed only in the bilateral Intermediate-subregion (pink; Figure 4A and Figure 4C).

#### 3.2.6 | Whole-brain metabolic covariance pattern (networks) of each hippocampal subregion

We examined the metabolic covariance pattern of each derived hippocampal subregion bilaterally across the subsample of healthy older subjects with high image resolution data using a univariate general linear model approach looking at the main effect pattern. Whole-brain metabolic networks of each hippocampal subregion with the significant brain regions labelled according to the Schaefer cortical and Melbourne subcortical functional atlases are presented in Figure 5A and Figure 5B. Overall, the anterior-subiculum (red) show primarily metabolic covariance with the prefrontal and temporal regions as well as the insula, extra-striate inferior cortex, the lateral amygdala, and the putamen suggesting a network corresponding to input information from anterior regions into the hippocampal head. In contrast, the posterior-subiculum (blue) showed the widest brain metabolic covariance pattern overlapping with most of the seven Yeo functional brain networks (Figure 5B). The posterior-subiculum (blue) seems to be part of an extended cortical system including several regions of the default mode, dorsal attention, ventral attention, and somatomotor networks. The intermediate-subregion (pink) was only significantly associated with the hippocampus head and body. The anterior-CA (yellow) was mainly bilaterally associated with the amygdala, while the posterior-CA (green) in the bilateral hippocampus was mainly associated with thalamus nuclei (Figure 5A and Figure 5B). In Figure 5C, we illustrated white matter tracts in medial temporal lobe including the uncinate fasciculus, fornix, and cingulum bundle tracts being created in the recent tractography studies.^58,59^ Structural connectivity studies have shown that the anterior end of the temporal lobe is connected to the frontal lobe, in particular, to the orbitofrontal cortex through the uncinate fasciculus white matter tract^59^ (Figure 5C). The fornix path plays a significant role in interconnecting the hippocampal formation with the subcortical structures (e.g., the post-commissural columns part of the fornix principally connects the hippocampal formation with the thalamus and hypothalamic nuclei^25^). Finally, the cingulum bundle path interconnects the output pathways from the hippocampus mainly originating directly from the subiculum to the ventral retro-splenial cortex, cingulate cortices, prelimbic, infralimbic, and frontal areas^60,61^ (Figure 5C).

#### 3.2.7 | Behavioral characterization of hippocampal metabolic networks

After identifying the metabolic organization of the hippocampus, we examined how the pattern of metabolic covariance of each hippocampal metabolic subregion relates to behavioral term-based meta-analytic maps. Only four metabolic networks (those of the anterior-subiculum [red], posterior-subiculum [blue], anterior-CA [yellow], and intermediate-subregion [pink]) show some resemblance with meta-analytic activation map of specific behavior term. The spatial pattern of anterior-subiculum (red) was primarily associated with terms pertaining to object and face recognition, language comprehension, semantics, and perception (Figure 6A). In contrast, the extended cortical network of the posterior-subiculum (blue) subregion appeared to be engaged in high-level goal-driven (“top-down”) cognition and action including terms such as working memory, cognitive demands, action, and spatial cognition (Figure 6A). As could be expected the metabolic network of the anterior-CA (yellow) was only associated with emotional and automated behavioral systems terms such as fear, happiness, conditioning, emotional, negative, and fearful (Figure 6A). Finally, the metabolic network of the intermediate-subregion (pink) was only associated with body-part terms such as limb, foot, and arm.

#### 3.2.8 | Neurotransmitter characterization of hippocampal metabolic networks

The metabolic networks of subiculum subregions (anterior-subiculum [red] and posterior-subiculum [blue]) interact with the serotonergic system with the 5HT1a, 5HT1b, and 5HT2a serotonergic receptors in addition to GABAA receptors (Figure 6B). In contrast, the metabolic networks of both the anterior and posterior CA subregions (anterior-CA [yellow] and posterior-CA [green]), specially the metabolic network of anterior-CA (yellow) subregion, overlap with the dopaminergic and serotonergic transporter systems (Figure 6B). Finally, the metabolic covariance pattern of the intermediate-subregion (pink) did not show any significant relationship with the spatial pattern of different neurotransmitters.

#### 3.2.9 | AD-related genetic characterization of hippocampal metabolic networks

When exploring the association between the spatial pattern of brain metabolic covariance of each hippocampal metabolic subregion and the brain spatial map of AD-related gene expression, we used *post mortem* gene expression data from the AHBA as a reference.^54^ We identified the list of genes that belongs to enriched pathways of differentially expressed genes in AD hippocampi reported in Xu et al.^53^ to extract the spatial expression patterns of disrupted enriched pathways in AD hippocampi (Figure 6C). The association between these spatial expression patterns and hippocampal metabolic networks is illustrated in Figure 6C. Only the metabolic networks of subiculum subregions (anterior-subiculum [red] and posterior-subiculum [blue]) interact with all the disrupted enriched pathways in AD hippocampi. The patterns of metabolic covariance of both the anterior and posterior CA subregions (anterior-CA [yellow] and posterior-CA [green]), and intermediate-subregion (pink) did not show significant spatial overlap with the expression patterns of AD hippocampal pathology-related genes.

## Supplementary Material

Tables s1-s4

Supp

## RESEARCH IN CONTEXT

### 1. Systematic Review:

Literature searches using PubMed were conducted to identify original and review articles, using keywords including “hippocampus, metabolic covariance, parcellation, aging, and Alzheimer’s disease.” These articles suggested that the hippocampus shows heterogeneous patterns of microstructural features, as well as heterogeneous anatomical and functional connectivity and that different subregions can be identified accordingly. However, PubMed searches did not identify studies revealing hippocampal parcellation based on the metabolic covariance and hippocampal subregion’s metabolic alterations in Alzheimer’s disease.

### 2. Interpretation:

Our study provides new evidence that anterior versus posterior and cornu ammonis versus subiculum subregions can be differentiated based on brain metabolic covariance and further reveals the behavioral, molecular, and pathological aging profiles of hippocampal metabolic subregions and networks.

### 3. Future Directions:

Future studies should use a longitudinal design and amyloid beta/tau positron emission tomography markers to delineate the cascade of pathological events within the different hippocampal subnetworks and the relationship between changes in the local metabolism of hippocampal anterior and posterior subregions and changes in their functional connectivity in heathy and pathological aging populations.

## References

[1] Insausti R, Amaral DG. Hippocampal formation. In: Paxinos G, Mai JK, eds. The Human Nervous System. Academic Press; 2004:871–914.

[2] Marks SM, Lockhart SN, Baker SL, Jagust WJ. Tau and beta-amyloid are associated with medial temporal lobe structure, function, and memory encoding in normal aging. J Neurosci. 2017;37(12):3192–3201. 28213439 10.1523/JNEUROSCI.3769-16.2017 PMC5373113

[3] Hampel H Amyloid-beta and cognition in aging and Alzheimer’s disease: molecular and neurophysiological mechanisms. J Alzheimers Dis. 2013;33(Suppl 1):S79–S86. 22531423 10.3233/JAD-2012-129003

[4] Villain N, Fouquet M, Baron JC, Sequential relationships between grey matter and white matter atrophy and brain metabolic abnormalities in early Alzheimer’s disease. Brain. 2010;133(11):3301–3314. 20688814 10.1093/brain/awq203 PMC3291528

[5] Fischer FU, Wolf D, Fellgiebel A. Alzheimer’s disease neuroimaging I. Diaschisis-like association of hippocampal atrophy and posterior cingulate cortex hypometabolism in cognitively normal elderly depends on impaired integrity of Parahippocampal cingulum fibers. J Alzheimers Dis. 2017;60(4):1285–1294. 29036815 10.3233/JAD-170147

[6] Pasquini L, Rahmani F, Maleki-Balajoo S, Medial temporal lobe disconnection and hyperexcitability across Alzheimer’s disease stages. J Alzheimers Dis Rep. 2019;3(1):103–112. 31259307 10.3233/ADR-190121 PMC6597961

[7] Weiler M, Agosta F, Canu E, Following the spreading of brain structural changes in Alzheimer’s disease: a longitudinal, multimodal MRI study. J Alzheimers Dis. 2015;47(4):995–1007. 26401778 10.3233/JAD-150196

[8] Chang YL, Chen TF, Shih YC, Chiu MJ, Yan SH, Tseng WY. Regional cingulum disruption, not gray matter atrophy, detects cognitive changes in amnestic mild cognitive impairment subtypes. J Alzheimers Dis. 2015;44(1):125–138. 25190630 10.3233/JAD-141839

[9] Alzheimer’s Association Calcium Hypothesis Workgroup. Calcium hypothesis of Alzheimer’s disease and brain aging: a framework for integrating new evidence into a comprehensive theory of pathogenesis. Alzheimers Dement. 2017;13(2):178–182.e17. 28061328 10.1016/j.jalz.2016.12.006

[10] Hachinski V, Einhaupl K, Ganten D, Preventing dementia by preventing stroke: the Berlin Manifesto. Alzheimers Dement. 2019;15(7):961–984. 31327392 10.1016/j.jalz.2019.06.001 PMC7001744

[11] Khachaturian ZS. Editorial: the ‘Aducanumab Story’: will the last chapter spell the end of the ‘Amyloid Hypothesis’ or mark a new beginning? J Prev Alzheimers Dis. 2022;9(2):190–192. 35542989 10.14283/jpad.2022.36

[12] Willette AA, Modanlo N, Kapogiannis D. Alzheimer’s disease neuroimaging I. Insulin resistance predicts medial temporal hypermetabolism in mild cognitive impairment conversion to Alzheimer disease. Diabetes. 2015;64(6):1933–1940. 25576061 10.2337/db14-1507 PMC4439566

[13] Small SA, Schobel SA, Buxton RB, Witter MP, Barnes CA. A pathophysiological framework of hippocampal dysfunction in ageing and disease. Nat Rev Neurosci. 2011;12(10):585–601. 21897434 10.1038/nrn3085 PMC3312472

[14] Amunts K, Kedo O, Kindler M, Cytoarchitectonic mapping of the human amygdala, hippocampal region and entorhinal cortex: intersubject variability and probability maps. Anat Embryol (Berl). 2005;210(5–6):343–352. 16208455 10.1007/s00429-005-0025-5

[15] Palomero-Gallagher N, Kedo O, Mohlberg H, Zilles K, Amunts K. Multimodal mapping and analysis of the cyto- and receptorarchitecture of the human hippocampus. Brain Struct Funct. 2020;225(3):881–907. 31955294 10.1007/s00429-019-02022-4 PMC7166210

[16] Li X, Li Q, Wang X, Li D, Li S. Differential age-related changes in structural covariance networks of human anterior and posterior hippocampus. Front Physiol. 2018;9:518. 29867561 10.3389/fphys.2018.00518 PMC5954440

[17] Plachti A, Kharabian S, Eickhoff SB, Hippocampus co-atrophy pattern in dementia deviates from covariance patterns across the lifespan. Brain. 2020;143(9):2788–2802. 32851402 10.1093/brain/awaa222 PMC7523701

[18] Plachti A, Eickhoff SB, Hoffstaedter F, Multimodal parcellations and extensive behavioral profiling tackling the hippocampus gradient. Cereb Cortex. 2019;29(11):4595–4612. 30721944 10.1093/cercor/bhy336 PMC6917521

[19] Robinson JL, Salibi N, Deshpande G. Functional connectivity of the left and right hippocampi: evidence for functional lateralization along the long-axis using meta-analytic approaches and ultra-high field functional neuroimaging. Neuroimage. 2016;135:64–78. 27132046 10.1016/j.neuroimage.2016.04.022

[20] Poppenk J, Evensmoen HR, Moscovitch M, Nadel L. Long-axis specialization of the human hippocampus. Trends Cogn Sci. 2013;17(5):230–240. 23597720 10.1016/j.tics.2013.03.005

[21] Przezdzik I, Faber M, Fernandez G, Beckmann CF, Haak KV. The functional organisation of the hippocampus along its long axis is gradual and predicts recollection. Cortex. 2019;119:324–335. 31181420 10.1016/j.cortex.2019.04.015

[22] Genon S, Bernhardt BC, La Joie R, Amunts K, Eickhoff SB. The many dimensions of human hippocampal organization and (dys)function. Trends Neurosci. 2021;44(12):977–989. 34756460 10.1016/j.tins.2021.10.003 PMC8616840

[23] Chauveau L, Landeau B, Dautricourt S, la Sayette VD, Chetelat G, de Flores R. Differential effects of age and sex on medial temporal lobe networks revealed by longitudinal analyses across the adult lifespan. Alzheimers Dementia. 2022;18(suppl 5):e062208.

[24] Petersen RC, Aisen PS, Beckett LA, Alzheimer’s Disease Neuroimaging Initiative (ADNI): clinical characterization. Neurology. 2010;74(3):201–209. 20042704 10.1212/WNL.0b013e3181cb3e25 PMC2809036

[25] Senova S, Fomenko A, Gondard E, Lozano AM. Anatomy and function of the fornix in the context of its potential as a therapeutic target. J Neurol Neurosurg Psychiatry. 2020;91(5):547–559. 32132227 10.1136/jnnp-2019-322375 PMC7231447

[26] Ashleigh T, Swerdlow RH, Beal MF. The role of mitochondrial dysfunction in Alzheimer’s disease pathogenesis. Alzheimers Dement. 2023;19(1):333–342. 35522844 10.1002/alz.12683

[27] Tonnies E, Trushina E. Oxidative stress, synaptic dysfunction, and Alzheimer’s disease. J Alzheimers Dis. 2017;57(4):1105–1121. 28059794 10.3233/JAD-161088 PMC5409043

[28] Esteras N, Abramov AY. Mitochondrial calcium deregulation in the mechanism of beta-amyloid and Tau pathology. Cells. 2020;9(9):2135. 32967303 10.3390/cells9092135 PMC7564294

[29] Wang X, Wang W, Li L, Perry G, Lee HG, Zhu X. Oxidative stress and mitochondrial dysfunction in Alzheimer’s disease. Biochim Biophys Acta. 2014;1842(8):1240–1247. 24189435 10.1016/j.bbadis.2013.10.015 PMC4007397

[30] Jurcau MC, Andronie-Cioara FL, Jurcau A, The link between oxidative stress, mitochondrial dysfunction and neuroinflammation in the pathophysiology of Alzheimer’s disease: therapeutic implications and future perspectives. Antioxidants (Basel). 2022;11(11):2167. 36358538 10.3390/antiox11112167 PMC9686795

[31] Llado A, Tort-Merino A, Sanchez-Valle R, The hippocampal longitudinal axis-relevance for underlying tau and TDP-43 pathology. Neurobiol Aging. 2018;70:1–9. 29935415 10.1016/j.neurobiolaging.2018.05.035

[32] de Flores R, Wisse LEM, Das SR, Contribution of mixed pathology to medial temporal lobe atrophy in Alzheimer’s disease. Alzheimers Dement. 2020;16(6):843–852. 32323446 10.1002/alz.12079 PMC7715004

[33] Hansson O, Seibyl J, Stomrud E, CSF biomarkers of Alzheimer’s disease concord with amyloid-beta PET and predict clinical progression: a study of fully automated immunoassays in BioFINDER and ADNI cohorts. Alzheimers Dement. 2018;14(11):1470–1481. 29499171 10.1016/j.jalz.2018.01.010 PMC6119541

[34] Blennow K, Shaw LM, Stomrud E, Predicting clinical decline and conversion to Alzheimer’s disease or dementia using novel Elecsys Abeta(1–42), pTau and tTau CSF immunoassays. Sci Rep. 2019;9(1):19024. 31836810 10.1038/s41598-019-54204-z PMC6911086

[35] Routier A, Burgos N, Guillon J, Clinica: an open source software platform for reproducible clinical neuroscience studies. Front Neuroinform. 2021;15:689675. 34483871 10.3389/fninf.2021.689675 PMC8415107

[36] Samper-Gonzalez J, Burgos N, Bottani S, Reproducible evaluation of classification methods in Alzheimer’s disease: framework and application to MRI and PET data. Neuroimage. 2018;183:504–521. 30130647 10.1016/j.neuroimage.2018.08.042

[37] Evans AC, Collins DL, Mills SR, Brown ED, Kelly RL, Peters TM, ed. 3D Statistical Neuroanatomical Models from 305 MRI Volumes: 1993 IEEE Conference Record Nuclear Science Symposium and Medical Imaging Conference, San Francisco, CA, USA, 31 October-6 November 1993. IEEE; 1993.

[38] Ashburner J, Friston KJ. Unified segmentation. Neuroimage. 2005;26(3):839–851. 15955494 10.1016/j.neuroimage.2005.02.018

[39] Ashburner J A fast diffeomorphic image registration algorithm. Neuroimage. 2007;38(1):95–113. 17761438 10.1016/j.neuroimage.2007.07.007

[40] Zhong Q, Xu H, Qin J, Zeng LL, Hu D, Shen H. Functional parcellation of the hippocampus from resting-state dynamic functional connectivity. Brain Res. 2019;1715:165–175. 30910629 10.1016/j.brainres.2019.03.023

[41] Thomas BA, Cuplov V, Bousse A, PETPVC: a toolbox for performing partial volume correction techniques in positron emission tomography. Phys Med Biol. 2016;61(22):7975–7993. 27779136 10.1088/0031-9155/61/22/7975

[42] Schaefer A, Kong R, Gordon EM, Local-global parcellation of the human cerebral cortex from intrinsic functional connectivity MRI. Cereb Cortex. 2018;28(9):3095–3114. 28981612 10.1093/cercor/bhx179 PMC6095216

[43] Tian Y, Margulies DS, Breakspear M, Zalesky A. Topographic organization of the human subcortex unveiled with functional connectivity gradients. Nat Neurosci. 2020;23(11):1421–1432. 32989295 10.1038/s41593-020-00711-6

[44] Eickhoff SB, Stephan KE, Mohlberg H, A new SPM toolbox for combining probabilistic cytoarchitectonic maps and functional imaging data. Neuroimage. 2005;25(4):1325–1335. 15850749 10.1016/j.neuroimage.2004.12.034

[45] Desikan RS, Segonne F, Fischl B, An automated labeling system for subdividing the human cerebral cortex on MRI scans into gyral based regions of interest. Neuroimage. 2006;31(3):968–980. 16530430 10.1016/j.neuroimage.2006.01.021

[46] Bellec P, Rosa-Neto P, Lyttelton OC, Benali H, Evans AC. Multi-level bootstrap analysis of stable clusters in resting-state fMRI. Neuroimage. 2010;51(3):1126–1139. 20226257 10.1016/j.neuroimage.2010.02.082

[47] Arslan S, Ktena SI, Makropoulos A, Robinson EC, Rueckert D, Parisot S. Human brain mapping: a systematic comparison of parcellation methods for the human cerebral cortex. Neuroimage. 2018;170:5–30. 28412442 10.1016/j.neuroimage.2017.04.014

[48] Genon S, Li H, Fan L, The right dorsal premotor mosaic: organization, functions, and connectivity. Cereb Cortex. 2017;27(3):2095–2110. 26965906 10.1093/cercor/bhw065 PMC5963821

[49] Genon S, Reid A, Li H, The heterogeneity of the left dorsal premotor cortex evidenced by multimodal connectivity-based parcellation and functional characterization. Neuroimage. 2018;170:400–411. 28213119 10.1016/j.neuroimage.2017.02.034 PMC5555826

[50] Iglesias JE, Van Leemput K, Augustinack J, Bayesian longitudinal segmentation of hippocampal substructures in brain MRI using subject-specific atlases. Neuroimage. 2016;141:542–555. 27426838 10.1016/j.neuroimage.2016.07.020 PMC5026967

[51] Wang L, Miller JP, Gado MH, Abnormalities of hippocampal surface structure in very mild dementia of the Alzheimer type. Neuroimage. 2006;30(1):52–60. 16243546 10.1016/j.neuroimage.2005.09.017 PMC2853193

[52] Dukart J, Holiga S, Rullmann M, A tool for spatial correlation analyses of magnetic resonance imaging data with nuclear imaging derived neurotransmitter maps. Hum Brain Mapp. 2021;42(3):555–566. 33079453 10.1002/hbm.25244 PMC7814756

[53] Xu M, Zhang DF, Luo R, A systematic integrated analysis of brain expression profiles reveals YAP1 and other prioritized hub genes as important upstream regulators in Alzheimer’s disease. Alzheimers Dement. 2018;14(2):215–229. 28923553 10.1016/j.jalz.2017.08.012

[54] Hawrylycz M, Miller JA, Menon V, Canonical genetic signatures of the adult human brain. Nat Neurosci. 2015;18(12):1832–1844. 26571460 10.1038/nn.4171 PMC4700510

[55] Arnatkeviciute A, Fulcher BD, Fornito A. A practical guide to linking brain-wide gene expression and neuroimaging data. Neuroimage. 2019;189:353–367. 30648605 10.1016/j.neuroimage.2019.01.011

[56] Quackenbush J Microarray data normalization and transformation. Nat Genet. 2002;32(Suppl 1):496–501. 12454644 10.1038/ng1032

[57] Fulcher BD, Little MA, Jones NS. Highly comparative time-series analysis: the empirical structure of time series and their methods. JR Soc Interface. 2013;10(83):20130048. 10.1098/rsif.2013.0048 PMC3645413 23554344

[58] Brown CA, Johnson NF, Anderson-Mooney AJ, Development, validation and application of a new fornix template for studies of aging and preclinical Alzheimer’s disease. Neuroimage Clin. 2017;13:106–115. 27942453 10.1016/j.nicl.2016.11.024 PMC5137184

[59] Archer DB, Moore EE, Shashikumar N, Free-water metrics in medial temporal lobe white matter tract projections relate to longitudinal cognitive decline. Neurobiol Aging. 2020;94:15–23. 32502831 10.1016/j.neurobiolaging.2020.05.001 PMC7483422

[60] Agster KL, Burwell RD. Hippocampal and subicular efferents and afferents of the perirhinal, postrhinal, and entorhinal cortices of the rat. Behav Brain Res. 2013;254:50–64. 23872326 10.1016/j.bbr.2013.07.005 PMC3792719

[61] Bubb EJ, Metzler-Baddeley C, Aggleton JP. The cingulum bundle: anatomy, function, and dysfunction. Neurosci Biobehav Rev. 2018;92:104–127. 29753752 10.1016/j.neubiorev.2018.05.008 PMC6090091

