# Supplementary material for: Hippocampal metabolic subregions and networks: Behavioral, molecular, and pathological aging profiles
Source: Alzheimers Dement. Author manuscript; Available in PMC 2024 May 1. (PMC10698199; doi:10.1002/alz.13056)
Supplement: Tables s1-s4 [file NIHMS1944292-supplement-Tables_s1-s4.docx]

**Supplementary Tables**

**Supplementary Table 1.** Demographic data of all **^18^FDG-PET** data

| Samples | Sample size (n) | Mean age $\boldsymbol{\pm}$SD (age range) | Male sample | Mean age $\boldsymbol{\pm}$SD (age range) | Females sample | Mean age $\boldsymbol{\pm}$SD (age range) |
| --- | --- | --- | --- | --- | --- | --- |
| healthy older: | | | | | | |
|  | n = 266 | 74.3$\boldsymbol{\pm}$6.1  (56 - 89) | n = 137 | 75$\boldsymbol{\pm}$5.86  (60 - 89) | n = 129 | 73.5$\boldsymbol{\pm}$6.22  (56 - 88) |
| early MCI: | | | | | | |
|  | n = 285 | 71.2$\boldsymbol{\pm}$7.4  (55 - 89) | n = 157 | 71.8$\boldsymbol{\pm}$6.99  (55 - 89) | n = 128 | 70.4$\boldsymbol{\pm}$7.83  (55 - 89) |
| late MCI: | | | | | | |
|  | n = 156 | 72.4$\boldsymbol{\pm}$7.49  (55 - 92) | n = 83 | 73.5$\boldsymbol{\pm}$7.16  (56 - 92) | n = 73 | 71.07$\boldsymbol{\pm}$7.66  (55 - 87) |
| AD: | | | | | | |
|  | n = 139 | 74.5$\boldsymbol{\pm}$8.17  (55 - 91) | n = 80 | 75.7$\boldsymbol{\pm}$8.08  (55 - 91) | n = 59 | 73$\boldsymbol{\pm}$8.09  (55 - 91) |

MCI = Mild cognitive impairment; AD = Alzheimer’s Disease; SD = standard deviation; n = number of subjects.

**Supplementary Table 2. ADNI samples characteristics**

|  | healthy older (n = 266) | | Early MCI (n = 285) | | Late MCI (n = 156) | | AD (n = 139) | |
| --- | --- | --- | --- | --- | --- | --- | --- | --- |
|  | ${A\beta}^{-}t{Tau}^{-}{pTau}^{-}$ | ${A\beta}^{+}t{Tau}^{+}{pTau}^{+}$ | ${A\beta}^{-}t{Tau}^{-}{pTau}^{-}$ | ${A\beta}^{+}{tTau}^{+}{pTau}^{+}$ | ${A\beta}^{-}t{Tau}^{-}{pTau}^{-}$ | ${A\beta}^{+}t{Tau}^{+}{pTau}^{+}$ | ${A\beta}^{-}t{Tau}^{-}{pTau}^{-}$ | ${A\beta}^{+}t{Tau}^{+}{pTau}^{+}$ |
| **n**  **(female)** | 80  (39) | 23  (10) | 105  (54) | 52  (23) | 26  (16) | 72  (35) | 5  (1) | 86  (43) |
| **Age**  **(SD)** | 72.19  (5.61) | 77.3  (4.89) | 68.98  (7.15) | 74.06  (6.67) | 70.35  (8.15) | 73.32  (6.66) | 76.9  (12.51) | 74.41  (8.31) |
| **Education**  **(SD)** | 15.93  (2.96) | 16.83  (2.39) | 16.18  (2.64) | 15.38  (2.90) | 16.38  (2.70) | 16.74  (2.53) | 16.4  (2.7) | 15.23  (2.64) |
| **MMSE**  **(SD)** | 29.04  (1.16) | 29.09  (1.38) | 28.59  (1.56) | 27.63  (1.83) | 28.27  (1.51) | 27.03  (1.77) | 24.8  (10.84) | 22.99  (2.02) |
| ***APOE ϵ4* allele (%)** | 18.75% | 47.83% | 21.9% | 46.15% | 19.23% | 58.33% | 0% | 48.84% |
| **CSF^*^ Aβ_1-42_ (pg/mL)**  **(SD)** | 1522.6  (329.04) | 691.68  (146.24) | 1436.5  247.73) | 687.67  (159.62) | 1457  (263.61) | 703.88  (163.54) | 1424.2  (162.46) | 617.25  (159.94) |
| **CSF^*^** tTau **(pg/mL)**  **(SD)** | 188.31  (35.54) | 344.53  (61.03) | 189.14  (42.83) | 408.95  (135.47) | 185.70  (41.35) | 398.48  (108.39) | 204.06  (45.72) | 427.19  (143.56) |
| **CSF^*^** pTau **(pg/mL)**  **(SD)** | 16.45  (3.10) | 35.25  (7.92) | 16.33  (3.8) | 42.74  (15.31) | 16.16  (3.66) | 40.02  (12.08) | 16.76  (3.89) | 42.72  (15.15) |

^*^: Aβ_1-42_, total (T)-tau and phosphorylated (P)-tau were analyzed using the fully automated Roche Elecsys and cobas e 601 immunoassay analyzer system. For this study, we combined data from the ADNI datasets “UPENNBIOMK9_04_19_17.csv, UPENNBIOMK10_07_29_19.csv

, and UPENNBIOMK12_01_04_21.csv”.

We used pre-established cut-offes for CSF AD biomarkers.[1, 2] The cut-off for Aβ(1–42) CSF AD biomarkers which measured by using novel Elecsys CSF immunoassays optimised for concordance of CSF biomarkers with amyloid-PET visual read were defined as 977 pg/mL (Aβ(1–42),)[2]and the cut-offs for pTau and tTau CSF AD biomarkers were optimised for identification of AD patients versus normal controls in the ADNI populations by a sensitivity analysis.[1]The cut-offs identified were 24 pg/mL (pTau) and 266 pg/mL (tTau) in ADNI.[1]

Data are given in mean values and standard deviation, SD. MCI = Mild cognitive impairment; AD = Alzheimer’s Disease; n = number of subjects.

**Supplementary Table 3. Different PET scanner technical procedures manual for FDG imaging in ADNI cohort.**

| **Phase of study** | **PET scanner** | **Reconstruction Parameters: FDG** | | | | | | | |
| --- | --- | --- | --- | --- | --- | --- | --- | --- | --- |
|  |  | **Grid** | **Field of view (FOV)** | **Slice Thickness** | **Zoom** | **TRIM** | **Voxel size** | **Smoothing**  **Filter** | **All corrections** |
| **ADNI 2 & ADNI GO** | GE Discovery STE and VCT - 47 slice PET/CT scanners | $128 \times128$ | 256 mm | 3.27 mm | - | - | 2.0 mm | NONE | ‘On’ |
|  | GE Discovery ST - 47 slice PET/CT scanners | $128 \times128$ | 256 mm | 3.27 mm | - | - | 2.0 mm | NONE | ‘On’ |
|  | GE Discovery RX - 47 slice (LYSO) PET/CT scanners | $128 \times128$ | 256 mm | 3.27 mm | - | - | 2.0 mm | NONE | ‘On’ |
|  | GE Discovery LS - 35 slice (PET/CT) scanners | $128 \times128$ | 256 mm | 4.25 mm | - | - | 2.0 mm | NONE | ‘On’ |
|  | GE Advance - 35 slice PET scanners | $128 \times128$ | 256 mm | 4.25 mm | - | - | 2.0 mm | NONE | ‘On’ |
|  | Philips Gemini TF - 90 slice PET/CT scanners | $128 \times128$ | 256 mm | 2.0 mm | - | - | 2.0 mm | ‘SHARP’ | ‘On’ |
|  | Philips Gemini and Gemini GXL - 90 slice PET/CT scanners | $128 \times128$ | 256 mm | 2.0 mm | - | - | 2.0 mm | ‘SHARP’ | ‘On’ |
|  | Philips Allegro - 90 slice PET scanners | $128 \times128$ | 256 mm | 2.0 mm | - | - | 2.0 mm | ‘SHARP’ | ‘On’ |
|  | Philips Allegro | $128 \times128$ | - | - | - | - | 2.0 mm | - | - |
|  | Siemens ECAT Exact HR+ (BGO) 63-slice scanners | $128 \times128$ | - | - | 2.0 | - | - | NONE | ‘On’ |
|  | Siemens HRRT 207-slice scanners | $256\times256$ | - | 1.219 mm | - | - | 1.219 mm | 2mm Gaussian | ‘On’ |
|  | Siemens BioGraph mCT - 81 or 109 (TrueV) slice PET/CT scanners | $400\times400$ | - | ~2.027 mm | 2.0 | - | ~1.018 mm | NONE | ‘On’ |
|  | Siemens BioGraph TruePoint - 81 or 109 (TrueV) slice PET/CT scanners (Model 1093) (TRIM:Off) | $336\times336$ | - | ~2.027 mm | 2.0 | Off | ~1.015 mm | NONE | ‘On’ |
|  | Siemens BioGraph TruePoint - 81 or 109 (TrueV) slice PET/CT scanners (Model 1093) (TRIM:ON) | $168 \times168$ | - | ~2.027 mm | 2.0 | ON | ~2.03 mm | NONE | ‘On’ |
|  | Siemens BioGraph HiRes - 81 slice PET/CT scanners (Model 1080) | $168 \times168$ | - | 2mm | 2.0 | ON | ~2.031 mm | NONE | ‘On’ |
|  | Siemens BioGraph (LSO) 47-slice PET/CT scanners | $128 \times128$ | - | - | 2.0 | ON | - | NONE | ‘On’ |
|  | Siemens ECAT Exact (BGO) and Accel (LSO) 47-slice scanners | $128 \times128$ | - | - | 2.0 | - | - | NONE | ‘On’ |
| **ADNI 3** | GE Discovery 600, 610, 690, and 710 - PET/CT scanners | $192\times192\times47$ | 256 mm | 3.27 mm | - | - | 1.333 mm | NONE | ‘On’ |
|  | GE Discovery STE - PET/CT scanners | $128 \times128$ | 256 mm | 3.27 mm | - | - | 2.0 mm | NONE | ‘On’ |
|  | GE Discovery ST - PET/CT scanners | $128\times128\times47$ | 256 mm | 3.27 mm | - | - | 2.0 mm | NONE | ‘On’ |
|  | GE Discovery LS - 35 slice (PET/CT) scanners | $128\times128\times47$ | 256 mm | 4.25 mm | - | - | 2.0 mm | NONE | ‘On’ |
|  | GE Advance - 35 slice PET scanners | $128\times128\times47$ | 256 mm | 4.25 mm | - | - | 2.0 mm | NONE | ‘On’ |
|  | Philips Ingenuity TF - PET/CT scanners | $128\times128\times90$ | 256 mm | 2.0 mm | - | - | 2.0 mm | ‘SHARP’ | ‘On’ |
|  | Philips Gemini TF - PET/CT scanners | $128\times128\times90$ | 256 mm | 2.0 mm | - | - | 2.0 mm | ‘SHARP’ | ‘On’ |
|  | Philips Gemini and Gemini GXL - 90 slice PET/CT scanners | $128\times128\times90$ | 256 mm | 2.0 mm | - | - | 2.0 mm | ‘SHARP’ | ‘On’ |
|  | Philips Allegro - 90 slice PET scanners | $128\times128\times90$ | 256 mm | 2.0 mm | - | - | 2.0 mm | ‘SHARP’ | ‘On’ |
|  | Siemens BioGraph mCT - mCT TrueV PET/CT scanners | $400\times400\times81$ | - | ~2.027 mm | 2.0 | - | ~1.018 mm | NONE | ‘On’ |
|  | Siemens BioGraph TruePoint – and TruePoint TrueV PET/CT  scanners (Models 1093, 1094) | $336\times336\times81$ | - | ~2.027 mm | 2.0 | - | ~1.015 mm | NONE | ‘On’ |
|  | Siemens BioGraph HiRes - 81 slice PET/CT scanners (Model  1080) | $168\times168\times81$ | - | ~2.00 mm | 2.0 | - | ~2.03 mm | NONE | ‘On’ |
|  | Siemens ECAT Exact HR+ (BGO) 63-slice scanners | $128\times128\times63$ | - | - | 2.0 | - | - | NONE | ‘On’ |
|  | Siemens HRRT 207-slice PET-only scanners | $256\times256\times207$ | - | 1.219 mm | - | - | 1.219 mm | 2mm Gaussian | ‘On’ |
|  | Siemens BioGraph (LSO; Models 1023,1024) 47-slice PET/CT  scanners | $128\times128\times47$ | - | - | 2.0 | ON | - | NONE | ‘On’ |
|  | Siemens ECAT Exact (BGO) and Accel (LSO) 47-slice PET-only  scanners | $128\times128\times47$ | - | - | 2.0 | - | - | NONE | ‘On’ |
|  | Siemens PET Systems (HR+, ECAT EXACT, and ACCEL using V7.2.2 software) | $128 \times128$ | 155 mm | - | 2.5 | - | - | NONE | ‘On’ |
|  | Siemens PET Systems-Biograph | $128 \times128$ | - | - | 2.5 | ON | 2.12 mm | All pass | ‘On’ |
|  | Siemens PET Systems - Biograph HiRez | $168 \times168$ | - | - | 2.0 | ON | - | All pass | ‘On’ |
|  | GE Advance/ GE Discovery LS | $128 \times128$ | 256 mm | - | - | - | - | NONE | ‘On’ |
|  | GE Discovery LST | $128 \times128$ | 256 mm | - | - | - | - | NONE | ‘On’ |
|  | Philips Allegro/Gemini | - | 256 mm | - | - | - | - | - | ‘On’ |

**Supplementary Table 4. Hub genes of the transcriptomic network that up-regulated and down-regulated AD-related genes reported in Xu et al. [3].**

| **Number** | **Gene** | **Upstream regulator^9^** | **Number** | **Gene** | **Upstream regulator^9^** | **Number** | **Gene** | **Upstream regulator*** |
| --- | --- | --- | --- | --- | --- | --- | --- | --- |
| 1 | *ADD3* | no | 31 | *GLTP* | no | 61 | *STXBP1* | no |
| 2 | *AGT* | yes | 32 | *NDE1* | yes | 62 | *SYT1* | no |
| 3 | *ATP1A2* | yes | 33 | *FA2H* | no | 63 | *TUBA4A* | no |
| 4 | *EPS8* | no | 34 | *TJAP1* | yes | 64 | *UCHL1* | yes |
| 5 | *GJA1* | yes | 35 | *CLIC1* | no | 65 | *YWHAB* | no |
| 6 | *HDAC1* | yes | 36 | *COL1A2* | no | 66 | *YWHAZ* | no |
| 7 | *HSPB1* | yes | 37 | *IL4R* | no | 67 | *RBM10* | no |
| 8 | *MSN* | yes | 38 | *OGN* | no | 68 | *SLC25A12* | no |
| 9 | *NOTCH2* | no | 39 | *STAT3* | no | 69 | *PEX11B* | no |
| 10 | *PON2* | yes | 40 | *TIMP1* | no | 70 | *INA* | no |
| 11 | *SOX9* | no | 41 | *TNFRSF1A* | no | 71 | *SNAP91* | no |
| 12 | *SSPN* | no | 42 | *IFITM3* | no | 72 | *CAP2* | no |
| 13 | *PRDX6* | no | 43 | *IFITM2* | no | 73 | *MLLT11* | no |
| 14 | *YAP1* | yes | 44 | *AMPH* | no | 74 | *STMN2* | yes |
| 15 | *FERMT2* | no | 45 | *ATP6V1B2* | no | 75 | *GHITM* | no |
| 16 | *PBXIP1* | no | 46 | *ATP6V1C1* | no | 76 | *TAGLN3* | no |
| 17 | *SLC31A2* | no | 47 | *ATP6V1E1* | no | 77 | *RAPGEFL1* | no |
| 18 | *LPAR1* | no | 48 | *DDX1* | no | 78 | *REEP1* | yes |
| 19 | *ERBB3* | no | 49 | *ENO2* | no | 79 | *VCAN* | no |
| 20 | *HSPA2* | yes | 50 | *GABRG2* | no | 80 | *CTNNA1* | no |
| 21 | *MAL* | no | 51 | *GLRB* | no | 81 | *SMAD5* | no |
| 22 | *KLK6* | yes | 52 | *GOT1* | no | 82 | *SP1* | no |
| 23 | *SOX10* | no | 53 | *GUCY1B3* | no | 83 | *SYPL1* | no |
| 24 | *TF* | no | 54 | *PCMT1* | no | 84 | *TJP1* | no |
| 25 | *UGT8* | no | 55 | *PFN2* | no | 85 | *TYK2* | no |
| 26 | *VEZF1* | no | 56 | *SERPINI1* | no | 86 | *SNAP23* | no |
| 27 | *ST18* | no | 57 | *MAPK9* | no | 87 | *IQGAP1* | no |
| 28 | *RASSF2* | no | 58 | *PSMD1* | no | 88 | *KAT2B* | no |
| 29 | *SLC44A1* | yes | 59 | *SCG5* | no | 89 | *BBX* | no |
| 30 | *DAAM2* | no | 60 | *SH3GL2* | yes |  |  |  |

^*^ Upstream regulator[3]: hub genes showed consistent early expression alterations in Mouseac [4] or in other two replicating datasets (GSE29317 [5] and GSE31372). 17 Genes highlighted in green are candidate upstream regulators, as indicated by early alteration.

Reference:

1. Blennow K, Shaw LM, Stomrud E, Mattsson N, Toledo JB, Buck K, et al. Predicting clinical decline and conversion to Alzheimer's disease or dementia using novel Elecsys Abeta(1-42), pTau and tTau CSF immunoassays. Sci Rep. 2019;9(1):19024.

2. Hansson O, Seibyl J, Stomrud E, Zetterberg H, Trojanowski JQ, Bittner T, et al. CSF biomarkers of Alzheimer's disease concord with amyloid-beta PET and predict clinical progression: A study of fully automated immunoassays in BioFINDER and ADNI cohorts. Alzheimers Dement. 2018;14(11):1470-81.

3. Xu M, Zhang DF, Luo R, Wu Y, Zhou H, Kong LL, et al. A systematic integrated analysis of brain expression profiles reveals YAP1 and other prioritized hub genes as important upstream regulators in Alzheimer's disease. Alzheimers Dement. 2018;14(2):215-29.

4. Matarin M, Salih DA, Yasvoina M, Cummings DM, Guelfi S, Liu W, et al. A genome-wide gene-expression analysis and database in transgenic mice during development of amyloid or tau pathology. Cell reports. 2015;10(4):633-44.

5. Kurronen A, Pihlaja R, Pollari E, Kanninen K, Storvik M, Wong G, et al. Adult and neonatal astrocytes exhibit diverse gene expression profiles in response to beta amyloid ex vivo. World J Neurosci. 2012;2(2):57-67.
